# Integrated Chemical Profiling and Network Pharmacology Establish a Quality Evaluation Framework for the Four Medicinal Parts of *Wolfiporia extensa* (Peck) Ginns

**DOI:** 10.3390/life16081241

**Published:** 2026-07-27

**Authors:** Shun-Xin Deng, Lih-Geeng Chen, Shih-Yi Hsiung, Vinh Tuyen T. Le, Chia-Jung Lee, Yves S. Y. Hsieh, Ching-Chiung Wang

**Affiliations:** 1 Ph.D. Program in Clinical Drug Development of Herbal Medicine, Taipei Medical University, Taipei 11031, Taiwan; d339107003@tmu.edu.tw (S.-X.D.); d339108003@tmu.edu.tw (V.T.T.L.); cjlee@tmu.edu.tw (C.-J.L.); yvhsieh@tmu.edu.tw (Y.S.Y.H.); 2 Department of Microbiology, Immunology and Biopharmaceuticals, College of Life Sciences, National Chiayi University, Chiayi 600355, Taiwan; lgchen@mail.ncyu.edu.tw; 3 School of Pharmacy, College of Pharmacy, Taipei Medical University, Taipei 11031, Taiwan; d301110009@tmu.edu.tw; 4 Department of Pharmacognosy—Traditional Pharmacy—Pharmaceutical Botany, College of Pharmacy, Can Tho University of Medicine and Pharmacy, Can Tho 941, Vietnam; 5 Graduate Institute of Pharmacognosy, Taipei Medical University, Taipei 11031, Taiwan; 6 Traditional Herbal Medicine Research Center, Taipei Medical University Hospital, Taipei 11031, Taiwan; 7 Division of Glycoscience, Department of Chemistry, School of Engineering Sciences in Chemistry, Biotechnology and Health, KTH Royal Institute of Technology, AlbaNova University Centre, SE10691 Stockholm, Sweden

**Keywords:** *Wolfiporia extensa* (WE), triterpenoids, monosaccharides, quality control, chemometric analysis, neuroprotection, PA/PAB, L* values

## Abstract

*Wolfiporia extensa* (Peck) Ginns (WE) is a fungus widely used in Traditional Chinese Medicine. Its four medicinal parts, Poria (WP), Rubra Poria (RP), Poriae Cutis (PC), and Poria Cum Pini Radix (PPR), are prescribed for distinct therapeutic purposes; however, their quality standards and pharmacological differences remain unclear. This study established reliable quality markers and compared the neuroprotective activities of four medicinal parts of WE. Seven triterpenoids were quantified using validated high-performance liquid chromatography (HPLC) assays, while monosaccharide profiles were analyzed using 1-phenyl-3-methyl-5-pyrazolone-HPLC. The International Commission on Illumination L*a*b* colorimetry and chemometric models were used for authentication. The neuroprotective effects of aqueous extracts were assessed in SH-SY5Y, BV-2, and HOG cell models. Network pharmacology was used to predict compound–target pathway relationships. The content of poricoic acid A (PAA) was the highest in PC; the content of pachymic acid (PA) was the highest in RP; whereas dehydrotumulosic acid (DTUA) was enriched in WP. WP demonstrated the highest mannose and galactose content. The water extract of WP reduced H_2_O_2_-induced cytotoxicity in SH-SY5Y cells, whereas the PC extract alleviated L-α-lysophosphatidylcholine-induced injury in human oligodendroglioma cells. Network analysis identified PA, PAA, and poricoic acid B (PAB) as candidate bioactive compounds involved in neuroprotective effects. The PA/PAB ratios of ≥10, 1–9, and ≤1 were used to identify WP, RP, and PC, respectively. L* values of >80, 55–79, and <55 effectively discriminated WP, RP, and PC, respectively. This integrative study identified chemical and functional quality markers and demonstrated distinct neuroprotective profiles in different parts of WE, supporting their standardized and rational application in traditional medicine and nutraceuticals.

## 1. Introduction

*Wolfiporia extensa* (Peck) Ginns (WE) is a sclerotium-forming fungus of the family Polyporaceae that grows in association with pine roots. Its sclerotium has been widely used in Traditional Chinese Medicine (TCM) to treat edema, insomnia [1,2], palpitations [3], and anxiety [4,5]. Modern pharmacological studies have demonstrated diverse biological activities of WE, including anti-inflammatory, antioxidant, immunomodulatory, and neuroprotective effects, providing pharmacological support for several of its traditional applications. In traditional medicinal practice, the sclerotium is divided into four medicinal parts-Poria (WP), Rubra Poria (RP), Poriae Cutis (PC), and Poria Cum Pini Radix (PPR)-which are used for different clinical purposes. Although these medicinal parts originate from the same fungal source [6,7], they are traditionally considered to possess distinct therapeutic properties, particularly diuretic and mind-calming (“An-Shen”) functions [8]. However, the phytochemical basis underlying these functional distinctions remains poorly understood.

**Figure 1:**
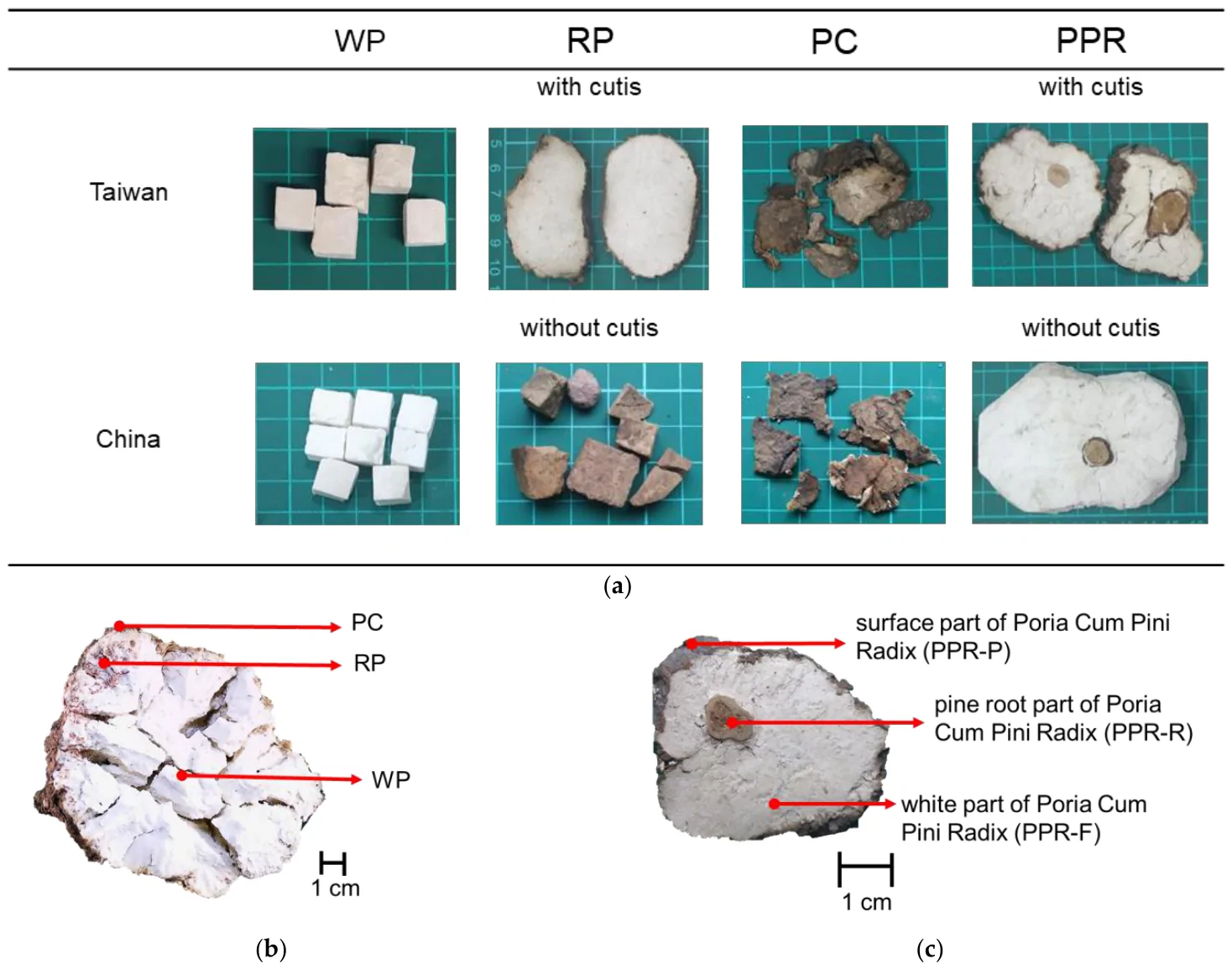
Appearance and schematic of *Wolfiporia extensa* (WE). (**a**) Appearance of WE in the Chinese and Taiwanese Pharmacopeia. (**b**) Schematic of the sclerotium. (**c**) Schematic of Poria Cum Pini Radix.

The classification and quality evaluation of the four medicinal parts of WE are inconsistent among official pharmacopeias and regional standards. The fourth edition of the Taiwan Herbal Pharmacopeia (THP4th) recognizes WP, RP, PC, and PPR as distinct medicinal parts [9], whereas the 2025 edition of the Chinese Pharmacopeia (ChP^2025^) includes only WP and PC as independent monographs [10]. RP and PPR are further defined differently in regional processing specifications [11,12]. In the THP4th, RP and PPR include the outer cutis, whereas the Chinese regional processing specifications define both materials as the reddish inner layer beneath the outer peel after removal of the cutis (Figure 1a). Despite this difference in botanical definition, both the Taiwanese and Chinese pharmacopeial systems classify RP as a dampness-draining medicinal material and PPR as a tranquilizing medicinal material. Such discrepancies indicate a lack of harmonized identification criteria and may contribute to inconsistencies in medicinal-part classification and quality assessment. Currently, differentiation of these medicinal parts relies largely on macroscopic and microscopic characteristics. These approaches are partly observer-dependent and become less reliable after the materials are processed into slices or powders. Objective chemical criteria are therefore needed to differentiate the four medicinal parts reliably.

Current pharmacopeial quality control of WE relies on a limited set of chemical markers, and the marker systems adopted vary among pharmacopeias. THP4th uses pachymic acid (PA) and polyporenic acid C (PPAC) as quality markers for different medicinal parts, whereas ChP^2025^ primarily employs β-1,3-glucan as a quality marker for WP [9,10]. Although these markers are used in routine quality control, they provide limited information on the phytochemical diversity among the four medicinal parts and do not adequately reflect their potentially distinct biological properties [9,10,13,14]. Therefore, a broader marker system that integrates discriminatory chemical characteristics with biological relevance may provide a more informative basis for identifying and evaluating the quality of WE medicinal parts.

Triterpenoids are major bioactive constituents of WE and are widely regarded as important chemical indicators for its quality assessment [13,14]. Structurally, WE triterpenoids are mainly classified into lanostane-, eburicane-, *seco*-lanostane-, and *seco*-eburicane-type compounds, several of which exhibit differential distribution within the sclerotium [13,14,15,16,17,18]. Advances in chromatographic and mass spectrometric techniques have facilitated the characterization of these constituents, while high-performance liquid chromatography (HPLC) remains a practical and reliable approach for routine quantitative analysis [19,20,21,22,23,24]. Based on previous phytochemical studies, structural diversity, and proposed biosynthetic relationships, seven representative triterpenoids-PA, PPAC, dehydrotumulosic acid (DTUA), dehydrotrametenolic acid (DTRA), dehydroeburicoic acid (DEA), poricoic acid A (PAA), and poricoic acid B (PAB) were selected for comparative analysis. Their distribution patterns may provide chemical signatures for distinguishing the four medicinal parts and may also contribute to their differential biological activities.

Among the traditional functions attributed to WE, the mind-calming effect is particularly relevant to neurological conditions associated with insomnia, neuroinflammation, and neurodegenerative processes [4,25,26,27,28,29]. Previous studies have demonstrated anti-inflammatory, antioxidant, and neuroprotective activities of WE extracts and individual triterpenoids [5,30,31,32,33,34,35,36,37,38,39,40,41,42]. However, most investigations have focused on whole sclerotium extracts or isolated compounds, and direct comparisons of the four traditional medicinal parts remain scarce. Consequently, whether their distinct chemical profiles are associated with differences in neuroprotective potential remains unclear. Integrating comparative phytochemical analysis with function-oriented biological evaluation may therefore facilitate the identification of markers that are useful not only for medicinal-part discrimination but also for reflecting their traditional functional characteristics.

Accordingly, this study aimed to develop an integrated chemical and biological strategy to evaluate the four traditional medicinal parts of WE. Seven representative triterpenoids were characterized and quantified using chromatographic and HPLC-based approaches to identify discriminatory chemical markers, and network pharmacology was employed as a hypothesis-generating approach to explore their potential neuroprotection-related targets and pathways. In clinical practice, WE is predominantly administered as a decoction. Given the limited water solubility of triterpenoids, aqueous extracts may more closely represent the chemical constituents encountered in traditional clinical use. Therefore, aqueous extracts of the four medicinal parts were subjected to comparative biological evaluation using complementary in vitro models of neuroinflammation, oxidative neuronal injury, and demyelination. Their monosaccharide compositions were also analyzed to explore potential associations between the chemical characteristics of the aqueous extracts and their biological activities. By integrating triterpenoid profiling, computational prediction, aqueous-extract characterization, and biological evaluation, this study aimed to identify discriminatory chemical characteristics and biologically relevant compositional features, thereby providing a scientific framework for differentiating, assessing quality, and standardizing the four medicinal parts of WE.

## 2. Materials and Methods

### 2.1. Materials

A total of 30 WP, 11 RP, 8 PC, and 9 PPR specimens were purchased from Chinese medicine stores in China and Taiwan (Table S1). Their appearance is shown in Figure 1a. Fresh WE sclerotia were harvested in Yunnan, China, in December 2018. The sclerotia were then separated into WP, RP, and PC parts and dried in an oven at 40 °C (Figure 1b). The separation of PPR sample is shown in Figure 1c. Samples were purchased from certified suppliers and authenticated based on compliance with pharmacopeial specifications. The samples were authenticated by Director Lih-Geeng Chen (Research Center for Chinese Herbal Medicine and Microbial Utilization, National Chiayi University) based on the morphological and chemical identification criteria specified in THP4th.

### 2.2. Nerve Cell Lines

The BV-2 cell line, a type of microglial cell line, was purchased from American Type Culture Collection (ATCC; Manassas, VA, USA); the SH-SY5Y cell line, a neuroblastoma cell line, was purchased from American Type Culture Collection (ATCC^®^ CRL-2266^TM^, Manassas, VA, USA) and Bioresource Collection and Research Center (BCRC 67018, Hsinchu, Taiwan). The HOG cell line, a human oligodendroglioma cell line, was purchased from Sigma-Aldrich (SCC163, Merck KGaA, Darmstadt, Germany).

### 2.3. Chemicals and Reagents

DMEM, DMEM/F12 (1:1), streptomycin and penicillin (PS), L-glutamine, and trypsin-EDTA were purchased from Gibco (Waltham, MA, USA). Fetal bovine serum was purchased from HyClone^TM^ (Cytiva, Marlborough, MA, USA). Six chemical reference substances (DTUA, PAA, PPAC, PA, DTRA, and DEA) were purchased from Chengdu Alfa Biotechnology (Chengdu, China). The PAB was isolated and characterized in our laboratory. Twelve monosaccharide standards, mannose (Man), ribose (Rib), rhamnose (Rha), N-acetylglucosamine (GlcNAc), glucuronic acid (GlcA), galacturonic acid (GalA), glucose (Glc), N-acetylgalactosamine (GalNAc), galactose (Gal), xylose (Xyl), arabinose (Ara), and fucose (Fuc), were purchased from Sigma-Aldrich (Darmstadt, Germany). The purity of each chemical reference substance and monosaccharide standard was greater than 98%. Methanol, trifluoroacetic acid (TFA), orthophosphoric acid (85%), and acetonitrile for high-performance liquid chromatography (HPLC) analysis were purchased from Merck (Darmstadt, Germany). Methanol was purchased from ECHO Chemicals (Miaoli, Taiwan). Deionized water was prepared using a Milli-Q water purification system (Millipore, Billerica, MA, USA). H_2_O_2_ was purchased from Honeywell Fluka (Muskegon, MI, USA). Lipopolysaccharide (LPS, O26:B6) and L-α-lysophosphatidylcholine (LPC) were purchased from Sigma-Aldrich (St. Louis, MO, USA).

### 2.4. Sample Preparation

The WE samples (WP, RP, PC, and PPR) were dried, pulverized, and passed through a 20-mesh sieve. Forty grams of powdered sample was extracted with distilled water at a solid-to-liquid ratio of 1:20 (*w*/*v*). The mixture was boiled until the water volume was reduced by half. The extracts were filtered through a Büchner funnel and freeze-dried. The freeze-dried extracts were used for nerve cell viability experiments and monosaccharide composition analyses.

One gram of sample powder was accurately weighed into a 15 mL centrifuge tube, 10 mL of methanol was added, and the mixture was ultrasonicated for 60 min. The solution was centrifuged at 3000 rpm for 10 min and filtered through a 0.45 µm polyvinylidene difluoride syringe filter. The filtrate was used as the sample solution for the HPLC analysis.

### 2.5. Cells Viability Assay

To comprehensively evaluate the neuroprotective potential of the aqueous extracts from the four medicinal parts of WE, three mechanistically relevant in vitro models were employed. SH-SY5Y human neuroblastoma cells were used as an oxidative stress model by treatment with H_2_O_2_ [43,44]; BV-2 murine microglial cells were stimulated with LPS to establish a neuroinflammatory model [45,46,47], and human oligodendroglioma (HOG) cells were treated with LPC to simulate demyelination-related injury associated with myelin disorders [48,49,50,51].

The BV-2 and HOG cell lines were maintained in the DMEM medium with 10% fetal bovine serum, 1% PS, and 1% L-glutamine at 37 °C in a 5% CO_2_ incubator, whereas the SH-SY5Y cells were maintained in the DMEM-F12 medium. Cells were seeded into 96-well plates and treated with 200 μg/mL aqueous extracts from the four medicinal parts (WP, RP, PC, and PPR) or the corresponding vehicle control. Oxidative stress was induced with 400 μM H_2_O_2_ in SH-SY5Y cells, neuroinflammation was induced with 1000 ng/mL LPS in BV-2 cells, and demyelination-related injury was induced with 50 μg/mL LPC in HOG cells. Following 24 h of co-treatment, cell viability was assessed using the 3-(4,5-dimethylthiazol-2-yl)-2,5-diphenyltetrazolium bromide (MTT) assay. Nitric oxide (NO) production in LPS-stimulated BV-2 cells was determined using the Griess reagent, and NO inhibition was calculated relative to the LPS-treated group. Cell viability was expressed as a percentage of the corresponding untreated control group. Cell viability assays were performed using independent biological experiments, each conducted in triplicate technical replicates.

### 2.6. Monosaccharide Composition Analysis of WE Samples

A total of 10 g of sample powder (five WP, six RP, six PC, and five PPR samples) was immersed in distilled water at 20× and boiled until the water volume was reduced to half of the original. Afterward, the extract was filtered and freeze-dried. The yields of the freeze-dried powder samples are listed in Table S4 (Supplementary Data). For polysaccharide hydrolysis, 22 water-extracted samples (10 mg each) of WP, RP, PC, and PPR were transferred into 4 mL vials. Next, 1 mL of 2 M TFA (Thermo Fisher Scientific, Waltham, MA, USA) was added to each vial. The samples were heated to 100 °C for 3 h. TFA was removed by heating for 30 min without caps, and the mixture was filtered after cooling.

For monosaccharide derivation with 1-phenyl-3-methyl-5-pyrazolone (PMP), 231 µL of the PMP reagent (0.5 M recrystallized PMP in methanol/1 M ammonium hydroxide: 20:18.5 *v*/*v*) was added to 60 µL of each TFA-hydrolyzed sample in a microcentrifuge tube. The microcentrifuge tubes were gently vortexed and heated at 70 °C for 1 h. The sample tubes were cooled on a bench for approximately 10 min. A total of 60 µL of 10 M formic acid was added to each sample tube. The samples were extracted four times with 850 µL of dibutyl ether (Thermo Fisher Scientific, Waltham, MA, USA) (shaken for 60 s, spun down, and the top layer was removed). The samples were concentrated using a SpeedVac (LAEYES) at 100 kPa to remove residual butyl ether. Next, the samples were centrifuged for 10 min at 20,800 RCF. Supernatants were transferred to HPLC vials.

An HPLC system (Waters Alliance e2695 Separations Module) was used for monosaccharide analysis. The column used was Phenomenex C18, 100 Å, 100 × 3 mm, 2.6 µm. Mobile phases were: (A) 10% acetonitrile in MQ water with 40 mM ammonium acetate; (B) 70% acetonitrile in MQ water. The elution was in a linear gradient: (B) mobile phase from 8 to 17% (0–30.0 min), 17–100% (30.0–30.4 min), 100–100% (30.4–43 min). Next, 8% of the (B) mobile phase was used to balance the column status for 5 min before the next sample injection. Man, Rib, Rha, GlcNAc, GlcA, GalA, Glc, GalNAc, Gal, Xyl, Ara, and Fuc were used as standards. Typical chromatograms are shown in Figure S1.

### 2.7. HPLC Analysis of Triterpenes in WE

Qualitative and quantitative analyses were performed using a Hitachi high-performance liquid chromatograph equipped with a 5110 pump, 5210 autosampler, and 5430 DAD (CM5000; Hitachi, Tokyo, Japan). Chromatographic separation was performed using a LiChrospher100 RP-18 end-capped column (4 mm internal diameter × 250 mm, 5 µm, Merck). The mobile phase contained 0.05% TFA in deionized water (A) and acetonitrile (B). PAB, DTUA, PAA, PPAC, DTRA, and DEA were analyzed after using the following linear gradient elution program: 0–5 min, 40–50% B; 5–30 min, 50–70% B; 30–50 min, 70–100% B. The column temperature was maintained at 40 °C. The flow rate was set at 1.0 mL/min. A 10 µL aliquot of the sample solution was injected for qualitative and quantitative analyses. The UV detector wavelength was set to 242 nm. Typical chromatograms are shown in Figure S2a.

PA was analyzed using a different mobile phase containing 0.1% phosphoric acid in deionized water (A) and acetonitrile (B). The mobile phase used the following linear gradient elution program: 0–20 min, 70–100% B; 21 min, 70% B; 21–30 min, 70% B. The column temperature was maintained at 40 °C. The flow rate was set to 1.0 mL/min. A 10 µL aliquot of the sample solution was injected for qualitative and quantitative analyses. The UV detector wavelength used was 203 nm. Typical chromatograms are shown in Figure S2b.

The repeatability was evaluated by analyzing six spiked sample (PC-1) solutions prepared in parallel. A recovery test was conducted using PC-1 with known amounts of the added chemical reference substances, along with six independent spiked analyses. The analytical method was validated in accordance with the ICH Q2(R2) guideline, with additional consideration of the recommendations provided in the Eurolab Technical Report. Considering the complexity of the WE samples and the concentration range of the analytes, an acceptance criterion of RSD < 10% was applied for the recovery test based on previously published studies [52].

Standard components were dissolved in methanol. A series of standard solutions was prepared by diluting the previous standard solution with methanol to achieve the desired concentration. A calibration curve was constructed by plotting the peak area (y-axis) against the standard solution concentration (x-axis). The linear concentrations used for different components were 15.6, 31.25, 62.5, 125, and 250 µg/mL for DTUA; 7.8, 15.6, 31.25, 62.5, and 125 µg/mL for PAA; 7.8, 15.6, 31.25, 62.5, 125, 250, and 500 µg/mL for PPAC; 7.8, 15.6, 31.25, 62.5, 125, and 250 µg/mL for PA; 15.6, 31.25, 62.5, 125, 250, and 500 µg/mL for DTRA and DEA; and 1.8, 3.9, 7.8, 15.6, 31.25, 62.5, 125, and 250 µg/mL for PAB. The limit of detection (LOD) and limit of quantification (LOQ) were calibrated as follows [53]:

(1) $$LOD=\frac{3\;\sigma }{S}$$

(2) $$LOD=\frac{10\;\sigma }{S}$$

where σ is the standard deviation of the response or the standard deviation of the y-intercepts, and S is the slope of the calibration curve.

### 2.8. Color Measurements

The color of each specimen was measured in five replicates and expressed using the CIE L*a*b* system with a color reader (CR-10; Konica Minolta, Osaka, Japan). The CIE L*a*b* system is a color space defined by the CIE in 1976 [54]. L* represents the lightness or black/white character of the color. a* and b* describe the chromatic characteristics of the colors. L* units, ranging from 0 (black) to 100 (white). The a* units range from 500 (red) to –500 (green), and the b* units range from 200 (yellow) to –200 (blue).

### 2.9. Data Analysis

Data preprocessing and latent Dirichlet allocation (LDA) analysis were performed in Python (version 3.9.0) using the scikit-learn, NumPy, pandas, and Matplotlib packages. Comparative analyses of different sample groups were performed using R (version 4.1.0), and boxplots were generated using the RColorBrewer package. Statistical analyses were performed using IBM SPSS Statistics version 19.0 (IBM Corp., Armonk, NY, USA). Differences among groups were evaluated using one-way analysis of variance (ANOVA), followed by Tukey’s honestly significant difference post hoc test for multiple comparisons. Statistical significance was defined as *p* < 0.05.

### 2.10. Network Pharmacology and Pathway Enrichment Analysis

A network pharmacology approach was used to explore the potential neuroprotective mechanisms of WE compounds. Nine representative compounds (PAB, DTUA, PAA, PA, PPAC, DTRA, DEA, Man, and Gal) were selected for target prediction. The chemical structures of these compounds were obtained from the PubChem database (https://pubchem.ncbi.nlm.nih.gov/, accessed on 18 July 2025), and their potential protein targets were predicted using the Swiss Target Prediction database (https://www.swisstargetprediction.ch/, accessed on 18 July 2025), with the organism restricted to *Homo sapiens* and the PubChem database. The GeneCards database (https://www.genecards.org/, accessed on 18 July 2025) was searched for genes related to neuroprotection using the keyword “neurodegeneration.” The compound target set and neuroprotection-related gene set were intersected to identify common targets, and the results were visualized using a Venn diagram (https://bioinformatics.psb.ugent.be/webtools/Venn/, accessed on 18 July 2025). The intersecting targets were considered candidates for the influence of WE on neuroprotective pathology. We analyzed the protein–protein interactions (PPI) among these intersecting targets using the STRING database (https://cn.string-db.org/, accessed on 18 July 2025). Conditions of use: the organism was *Homo sapiens*, and the clustering option was k-means.

The Kyoto Encyclopedia of Genes and Genomes (KEGG) pathway enrichment analysis was performed using the DAVID Bioinformatics Resource (https://davidbioinformatics.nih.gov/, accessed on 18 July 2025), and the top 15 enriched pathways were charted. Conditions of use: the organism was *Homo sapiens*. From these, we selected relevant signaling pathways and examined WE compounds associated with proteins in these pathways, summarizing the compound–target–pathway relationships in a heatmap.

## 3. Results

### 3.1. Neuroprotective Effects of Four Medicinal Parts of WE in Neuron Cells

**Figure 2:**
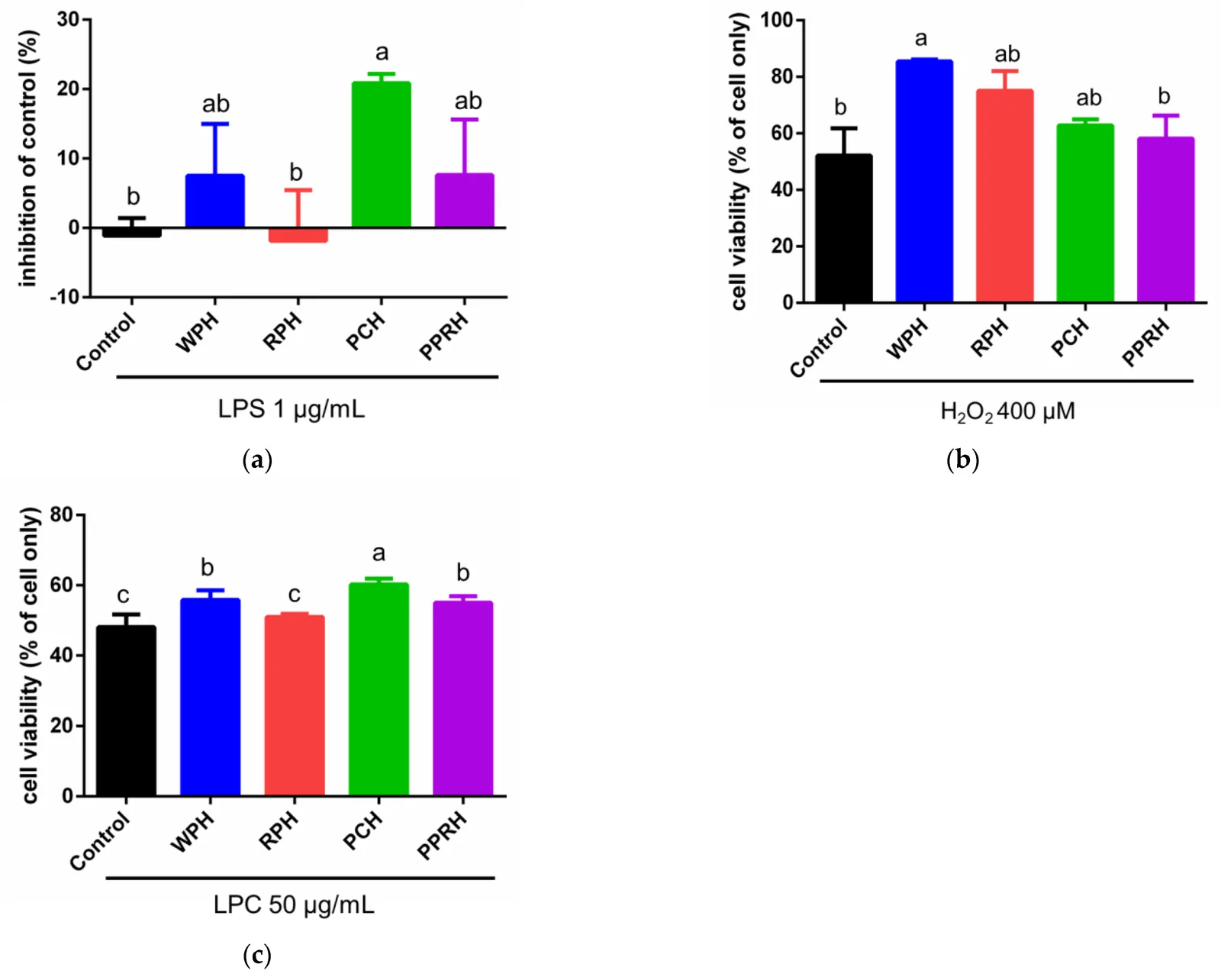
Neuroprotective effects of four medicinal parts of WE. (**a**) NO inhibition rate in LPS−induced BV−2 cells. (**b**) Cell viability of H_2_O_2_−induced SH−SY5Y cells. (**c**) Cell viability of LPC−induced HOG cells. WPH, aqueous extract of Poria (blue); RPH, aqueous extract of Rubra Poria (red); PCH, aqueous extract of Poriae Cutis (green); PPRH, aqueous extract of Poria Cum Pini Radix (purple). Different lowercase letters above the bars within the same picture indicate statistically significant differences among groups (one-way ANOVA followed by Tukey’s multiple comparison test, *p* < 0.05).

The inhibitory effects of the four aqueous extracts of WE (WPH, RPH, PCH, and PPRH) on NO production were measured in LPS-induced BV-2 cells. At a concentration of 200 μg/mL, all samples demonstrated less than 40% inhibition of NO production and exhibited no cytotoxicity. PC exhibited the most potent inhibitory effect (Figure 2a).

**Figure 3:**
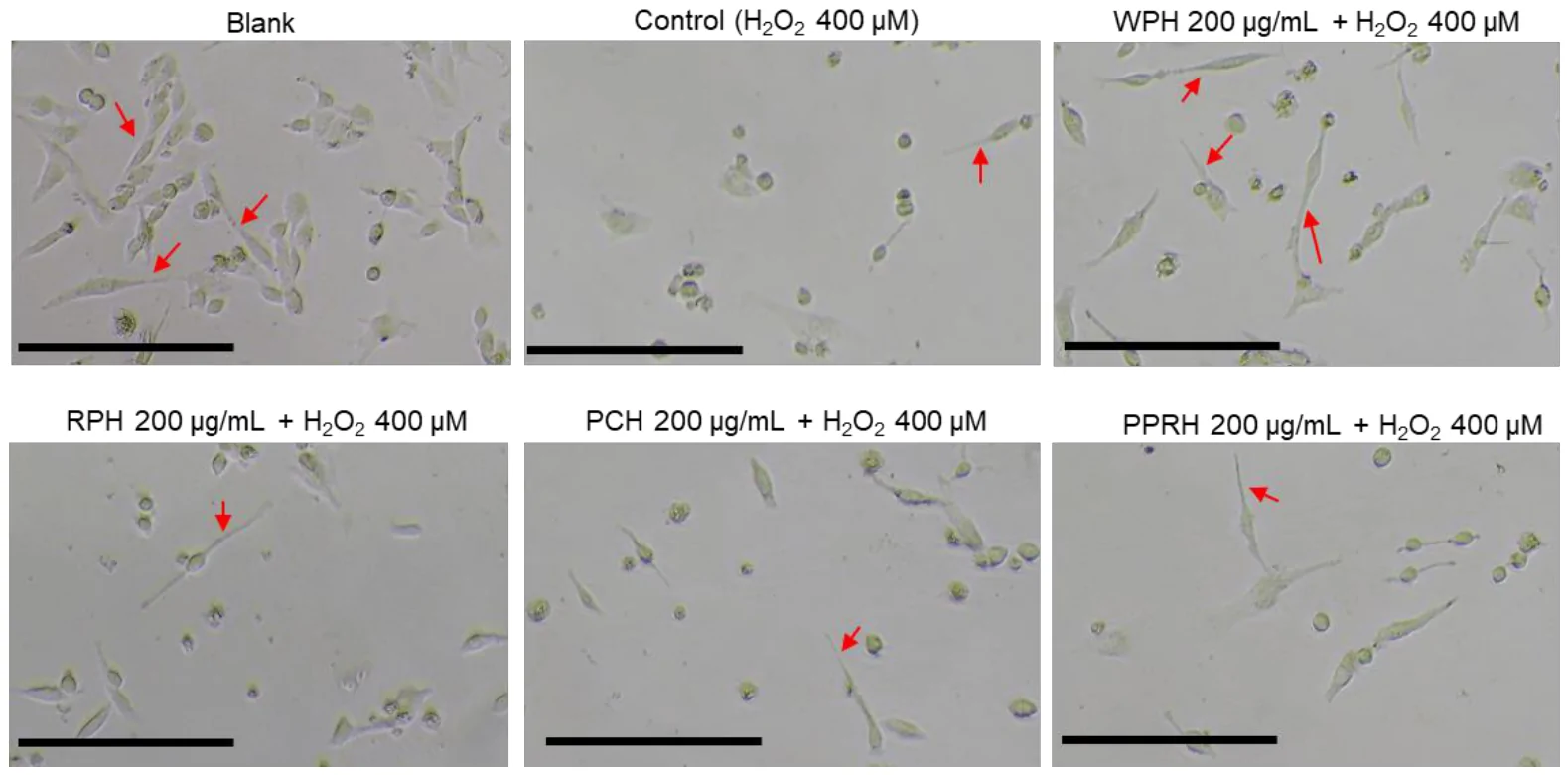
Cell morphology of four medicinal parts of WE in H_2_O_2_-induced SH-SY5Y cells. WPH, aqueous extract of Poria; RPH, aqueous extract of Rubra Poria; PCH, aqueous extract of Poriae Cutis; PPRH, aqueous extract of Poria Cum Pini Radix. Scale bar  =  100 µm. Red arrows indicate viable cells and their neurite-like processes.

Treatment with 400 μM H_2_O_2_ reduced SH-SY5Y cell viability to approximately 50% of the untreated control (Figure 2b). At 200 μg/mL, aqueous extracts of all four WE medicinal parts increased cell viability following H_2_O_2_ exposure. The relative effects were observed in the order WPH > RPH > PCH > PPRH, with the WP extract showing a significant increase in cell viability compared with the H_2_O_2_-treated group (Figure 2b). Morphological observation showed that H_2_O_2_-treated SH-SY5Y cells exhibited shortened neurite-like processes, whereas cells treated with the WE extracts showed less pronounced morphological alterations (Figure 3).

Treatment with 50 μg/mL LPC reduced HOG cell viability to approximately 50% of the untreated control (Figure 2c). At 200 μg/mL, RP and PPR extracts significantly increased the viability of LPC-treated HOG cells by approximately 10–15% compared with the LPC-treated group (Figure 2c).

### 3.2. Monosaccharide Content in WE Samples

**Figure 4:**
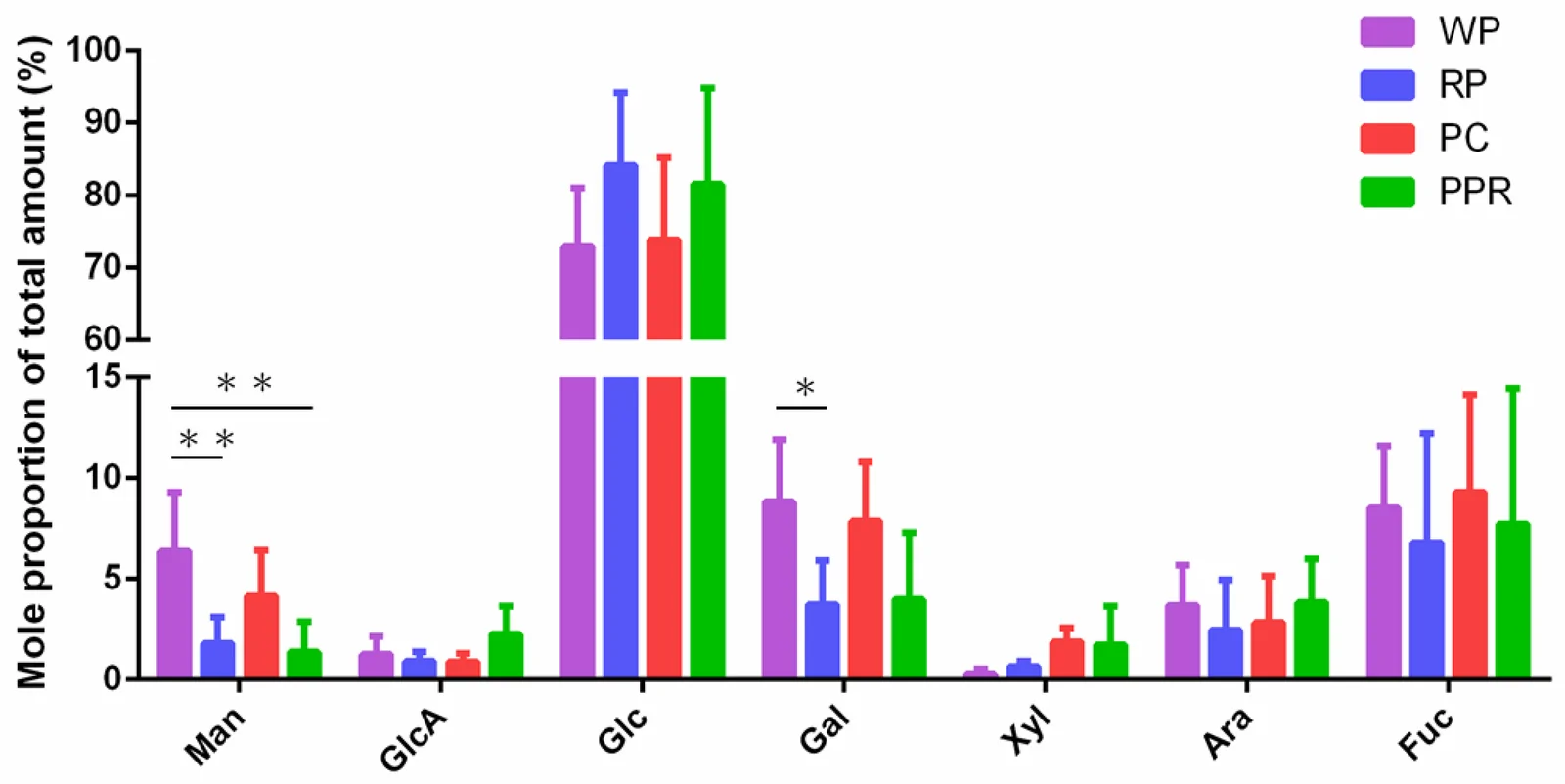
Monosaccharide composition and mole proportion (%) between the four medicinal parts of WE samples. *: *p* < 0.05 and **: *p* < 0.01. Man, mannose; GlcA, glucuronic acid; Glc, glucose; Gal, galactose; Xyl, xylose; Ara, arabinose; Fuc, fucose. WP, Poria (blue); RP, Rubra Poria (red); PC, Poriae Cutis (green); PPR, Poria Cum Pini Radix (purple). Mole proportion (%) = (mole content of each monosaccharide/total mole content of all detected monosaccharides) × 100.

Twelve monosaccharide standards were analyzed (Figure S1); however, only seven monosaccharides (Man, GlcA, Glc, Gal, Xyl, Ara, and Fuc) were detected in the WE. Five WP, six RP, six PC, and five PPR aqueous extract samples were selected to determine the monosaccharide content. The average mole proportion of Glc was 78.16% (59.58–95.54%) in WE; however, no significant differences were observed among the four medicinal parts (Table S5). A comparison of the monosaccharide composition and mole proportion between the four medicinal parts of WE displayed significant differences between Man and Gal. The largest molar proportions of Man and Gal were found in WP, and the lowest were found in RP (Figure 4).

### 3.3. Validation of the Quantitative HPLC Method

PAB, DTUA, PAA, PPAC, PA, DTRA, and DEA were used as the chemical indicators for WP, RP, PC, and PPR, respectively. All calibration curves exhibited high coefficients of determination (*r*^2^ > 0.9990) across a relatively wide concentration range. LOD ranged from 0.54 to 11.59 µg/mL, and LOQ ranged from 1.80 to 38.63 µg/mL (Table 1). The relative standard deviations (RSDs) were used to assess the method’s precision and repeatability. The intra- and inter-day precisions of seven compounds demonstrated RSDs of <3.61% and <3.79%, respectively. The RSDs of the stability assay were <5.0%. The recoveries of analytes were 93.86–109.98%, with 0.84–7.52% RSDs (Table 2). The highest RSD (7.52%) was observed only for DEA, whereas the remaining analytes exhibited substantially lower variability. These results demonstrated that the proposed quantitative HPLC methods are sufficiently linear, precise, sensitive, and repeatable for the simultaneous determination of seven chemical indicators in the four medicinal parts of WE.

### 3.4. Contents of Seven Chemical Indicators in WE

**Figure 5:**
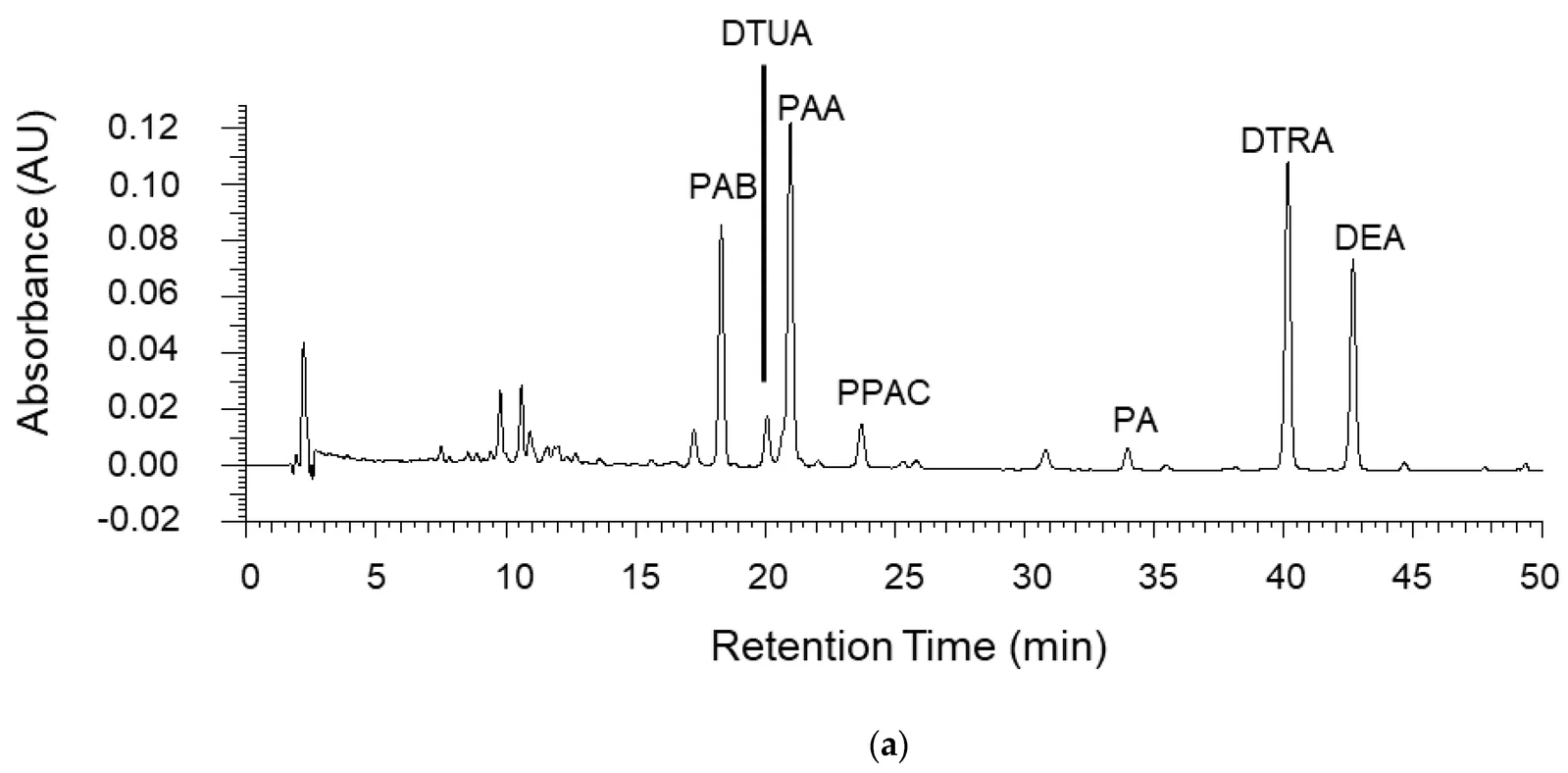

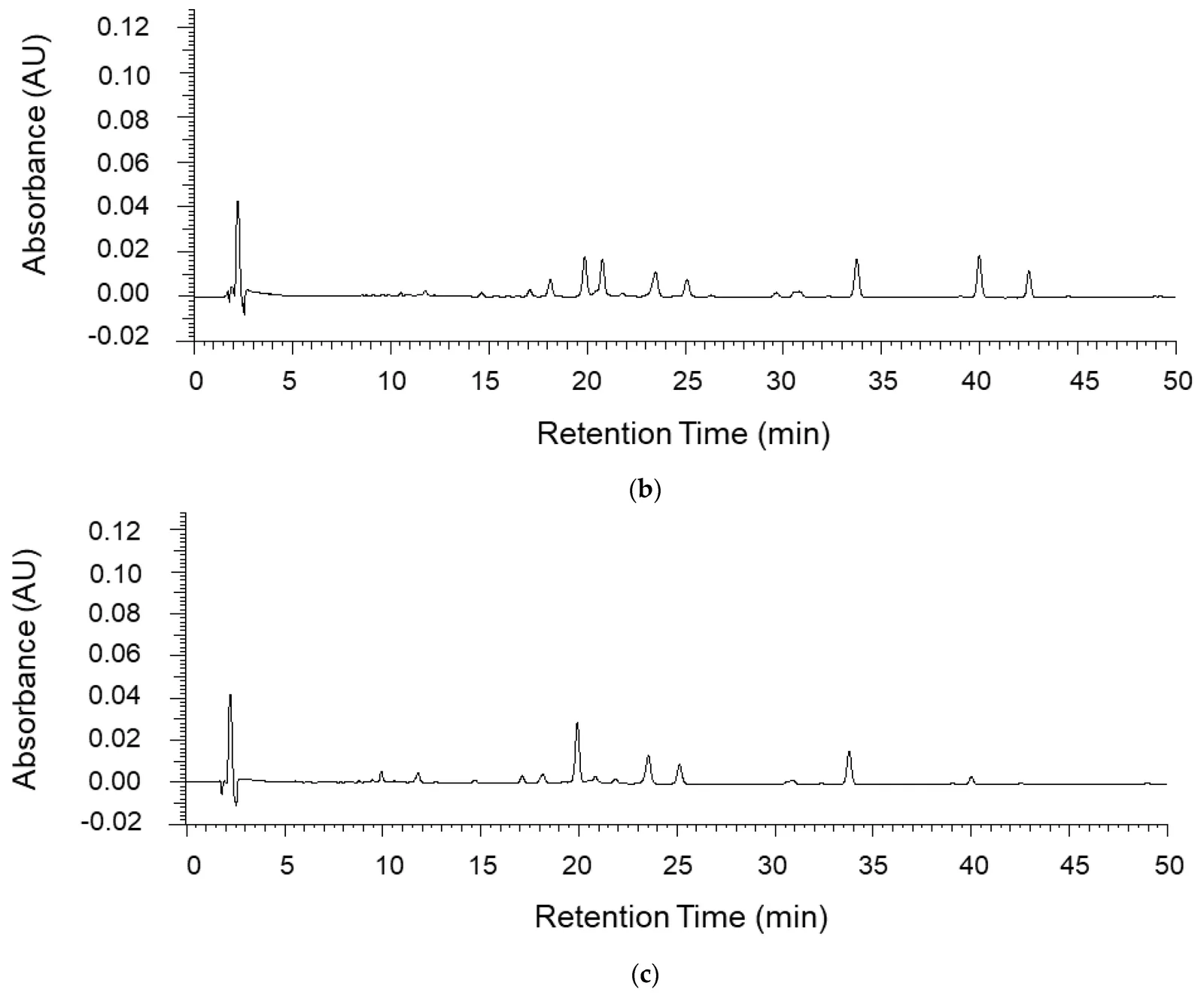
High−performance liquid chromatography (HPLC) chromatograms of different medicinal parts of WE samples. (**a**) Poriae Cutis (PC); (**b**) Rubra Poria (RP); (**c**) Poria (WP). Chromatograms of PAB, DTUA, PAA, PPAC, DTRA, and DEA of three medicinal parts. The UV detector was set to 242 nm.

**Figure 6:**
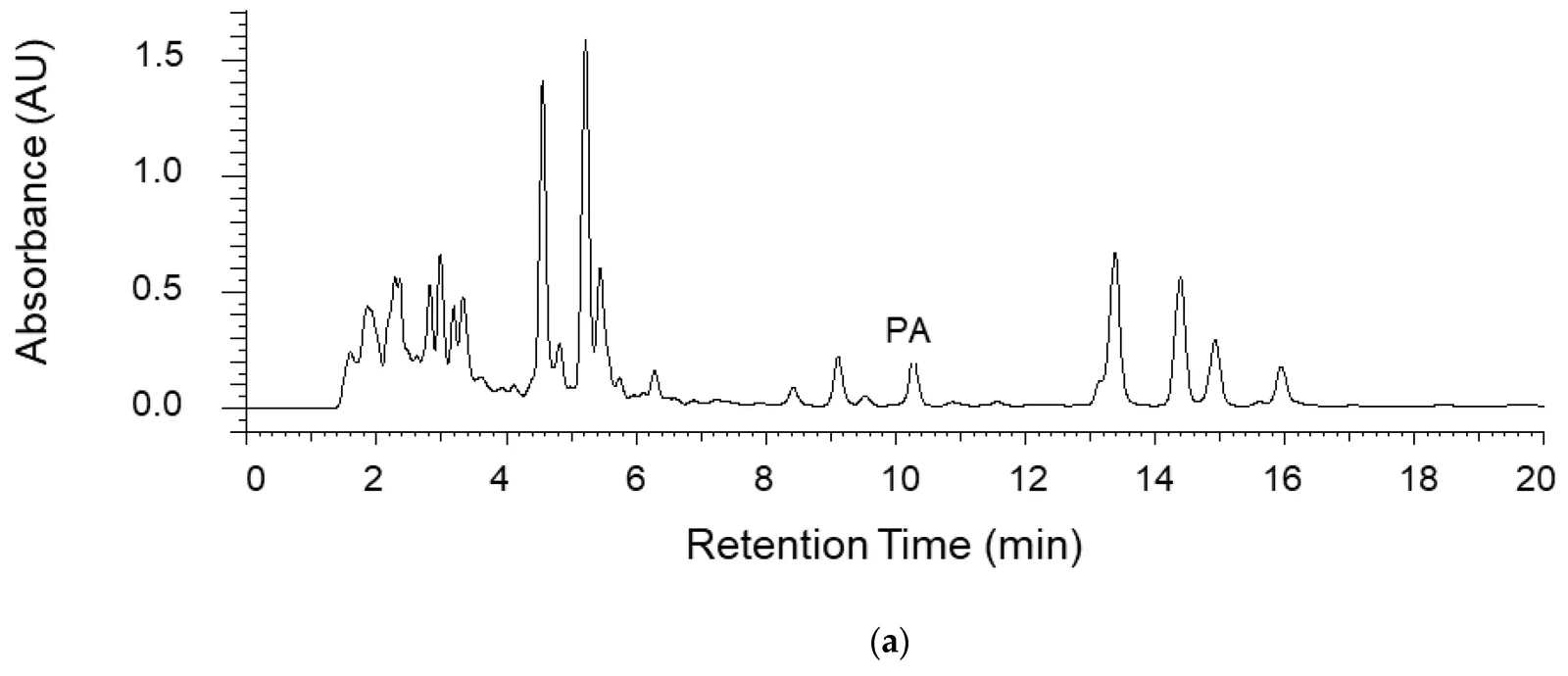

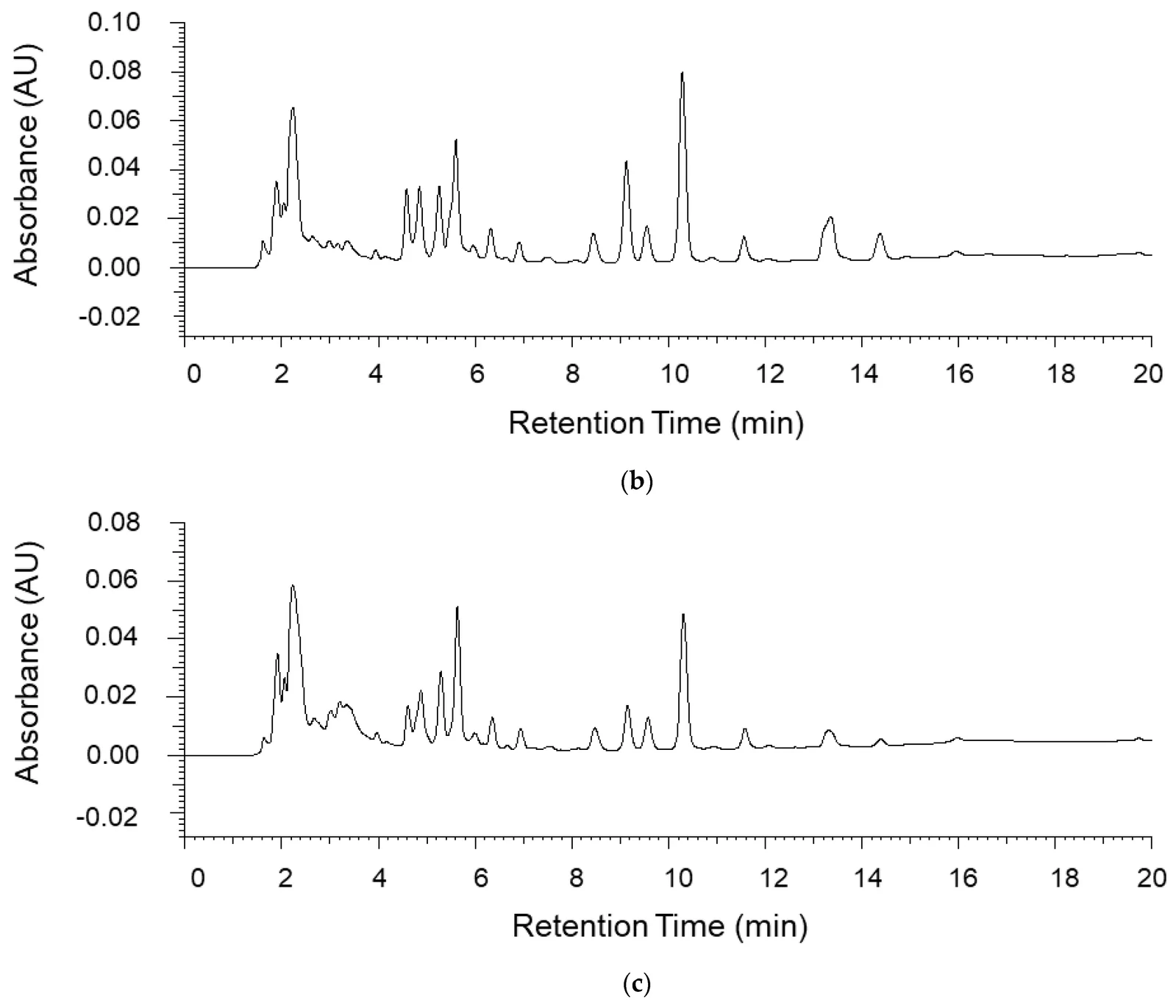
High−performance liquid chromatography (HPLC) chromatograms of different medicinal parts of WE samples. (**a**) Poriae Cutis (PC); (**b**) Rubra Poria (RP); (**c**) Poria (WP). Chromatograms of PA of three medicinal parts. The UV detector was set to 203 nm.

Because triterpenoids were present at low concentrations in the aqueous extracts, methanol extracts were used for HPLC quantification. Seven triterpenoid compounds naturally occurring in WE sclerotia were selected as chemical indicators and quantified in the three medicinal parts of WE sclerotia, namely WP, RP, and PC (Figure 1b). Representative HPLC chromatograms of the six chemical indicators (PAB, DTUA, PAA, PPAC, DTRA, and DEA) are shown in Figure 5. Since the PA peak was difficult to clearly identify under the conditions used for simultaneous analysis of the six chemical indicators, an optimized HPLC method was further applied for PA determination. The corresponding HPLC chromatogram of PA is shown in Figure 6. The contents of the total chemical indicators were in the order of PC > RP > WP. The contents of total chemical indicators in PC were considerably greater than those in RP and WP. PC had high PAA, DTRA, DEA, and PAB content at 11.55 ± 1.25 mg/g, 7.52 ± 0.96 mg/g, 5.99 ± 0.73 mg/g, and 7.21 ± 0.65 mg/g, respectively (Table 3).

### 3.5. Chemometric Prediction of Seven Chemical Indicators in Commercial Samples

**Figure 7:**
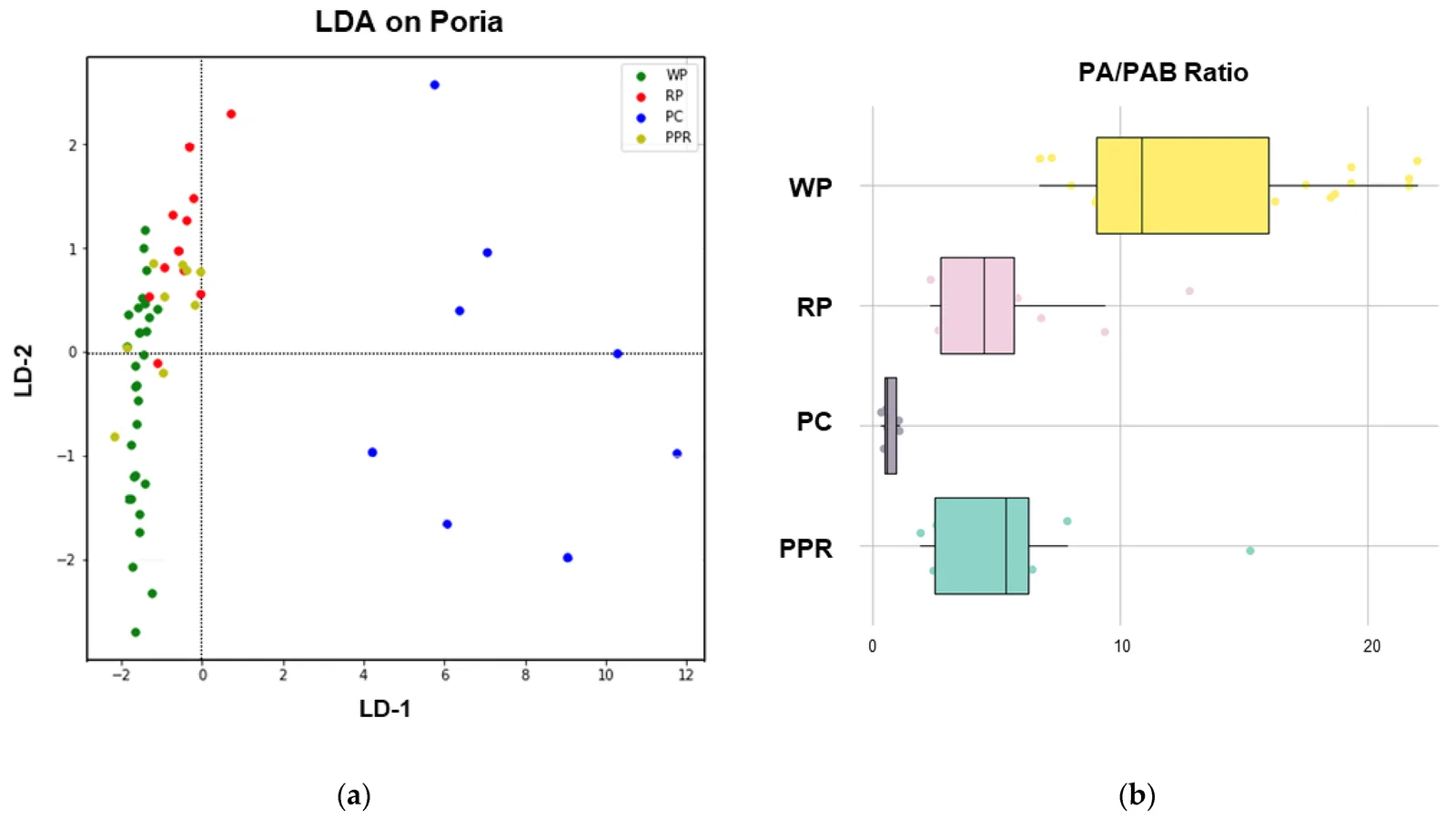
Seven reference substances of 58 WE samples. (**a**) Linear discriminant analysis of seven chemical indicators in four medicinal parts of WE, Poria (WP; green), Rubra Poria (RP; red), Poriae Cutis (PC; blue), and Poria Cum Pini Radix (PPR, yellow). (**b**) Boxplot of PA/PAB ratio in four medicinal parts of WE, WP (yellow), RP (pink), PC (gray), and PPR (green).

LDA was performed using seven representative triterpenoids to evaluate discrimination among the four medicinal parts of WE. The analysis included 30 WP, 11 RP, 8 PC, and 9 PPR samples. Among the RP samples, RP-1–RP-6 were collected from Taiwan and retained the cutis, whereas RP-7–RP-11 were obtained from China and lacked the cutis. The LDA score plot revealed three distinct clusters corresponding to WP, RP, and PC (Figure 7a). However, two RP samples (RP-6 and RP-7) were located within or adjacent to the WP cluster, indicating that their triterpenoid profiles were more similar to those of WP than to the remaining RP samples.

Higher contents of PAB, PAA, DTRA, and DEA were observed in PC samples. Among the seven quantified triterpenoids, PAA was the predominant compound in PC. The total triterpenoid content increased from the inner to the outer regions of WE. Among the 30 WP samples, WP-15 exhibited the highest total content of the seven quantified triterpenoids (Table S2). DTRA and DEA were detected in only six WP samples (WP-1, WP-5, WP-7, WP-8, WP-29, and WP-30), whereas DTUA and PA were the predominant triterpenoids in WP and RP, respectively.

The PA/PAB ratio differed markedly among the three medicinal parts, ranging from 6.75 to 34 in WP, 2.3–12.8 in RP, and 0.3–1.07 in PC (Figure 7b). Based on these distributions, PA/PAB ratios ≥10, 1–9, and ≤1 corresponded to WP, RP, and PC, respectively.

### 3.6. Colors of WE Samples

**Figure 8:**
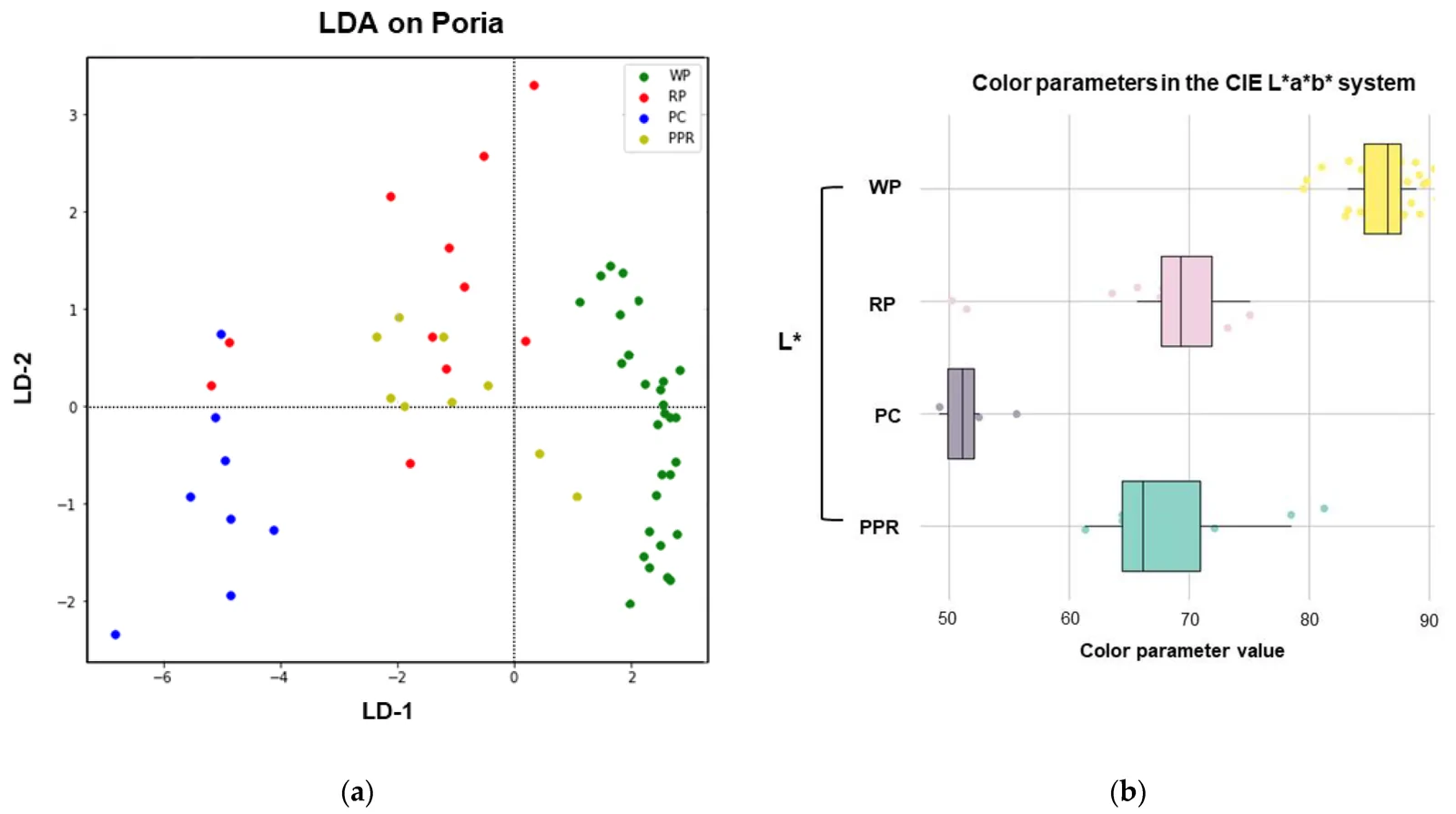
Color parameters of 58 WE samples. (**a**) Linear discriminant analysis of L*a*b* values in four medicinal parts of WE, Poria (WP, green), Rubra Poria (RP, red), Poriae Cutis (PC, blue), and Poria Cum Pini Radix (PPR, yellow). (**b**) Boxplot of L* values in four medicinal parts of WE, WP (yellow), RP (pink), PC (gray), and PPR (green).

Because the surface color of WE medicinal materials is often heterogeneous, color measurements were performed using powdered samples. Color parameters using the CIE L*a*b* system were analyzed by LDA from 30 WP, 11 RP, 8 PC, and 9 PPR samples. The analysis revealed three distinct clusters corresponding to WP, RP, and PC (Figure 8a). However, two RP samples (RP-9 and RP-11) overlapped with the PC samples. Among the color parameters, WP exhibited the highest L* values and negative a* values, whereas RP and PC showed lower L* values and positive a* values (Table S3). The L* values of WP, RP, and PC were 81.02–90.66, 50.24–75.04, and 44.22–55.62, respectively. Based on these distributions, L* values >80, 55–79, and <55 distinguished WP, RP, and PC, respectively (Figure 8b).

LDA analysis revealed two distinct clusters of RP samples (Figure 8a). RP-1 to RP-6 were collected from Taiwan and retained the cutis, whereas RP-7 to RP-11 were obtained from China and lacked the cutis. The Taiwanese RP samples exhibited L* values ranging from 64 to 76, while the Chinese samples ranged from 50 to 74. Among the five Chinese samples, three showed relatively low L* values (50–64), indicating greater color variation within this group. Partial overlap between RP and PC was observed in the LDA plot, particularly among the Chinese samples. In addition, after pulverization, the PC samples exhibited characteristic cotton-like fibrous structures that were not observed in the RP samples.

### 3.7. Contents of Seven Chemical Indicators and Their Colors in PPR Samples Obtained from Markets

Nine commercial PPR samples were purchased from China and Taiwan. With the exception of PPR-9, all market samples retained the cutis. HPLC analysis showed that the contents of the seven quantified triterpenoids were generally higher in the PPR samples than in the WP samples. In particular, PAA, PA, and DTRA were present at relatively higher levels (Table S2). In contrast, PPR-9 exhibited lower contents of PAA, DTRA, and DEA, but a higher PA content than the other PPR samples. The PA/PAB ratio ranged from 1.9 to 15.2 (Figure 7b), which overlapped with the range observed for RP. The L* values of the PPR samples ranged from 61 to 79 (Table S3 and Figure 8b). Among them, PPR-9 showed the highest L* value, consistent with the absence of the cuticle. Overall, the PA/PAB ratio and L* values of the PPR samples largely overlapped with those of RP.

### 3.8. Contents of Chemical Indicators in Different Parts of PPR

**Figure 9:**
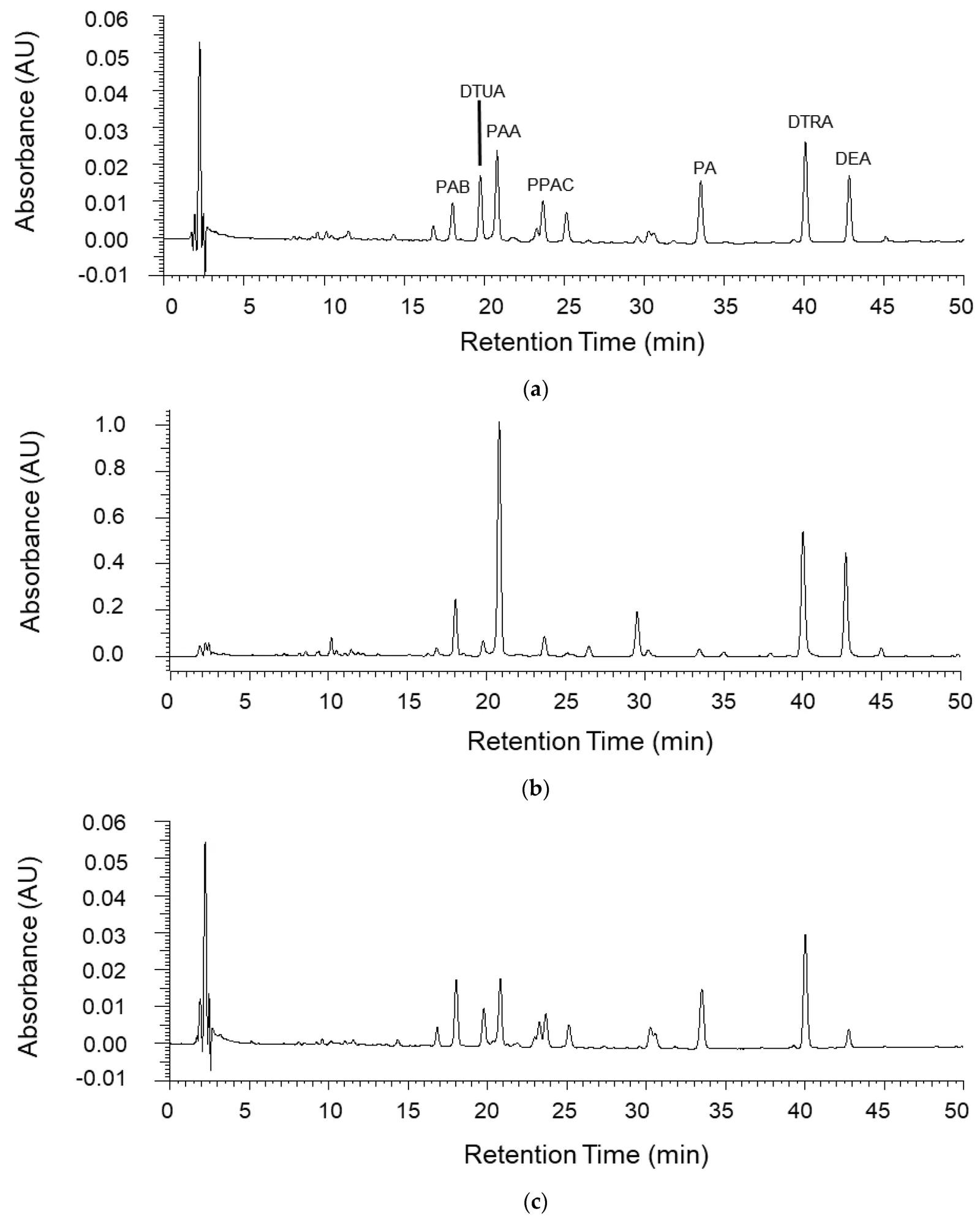

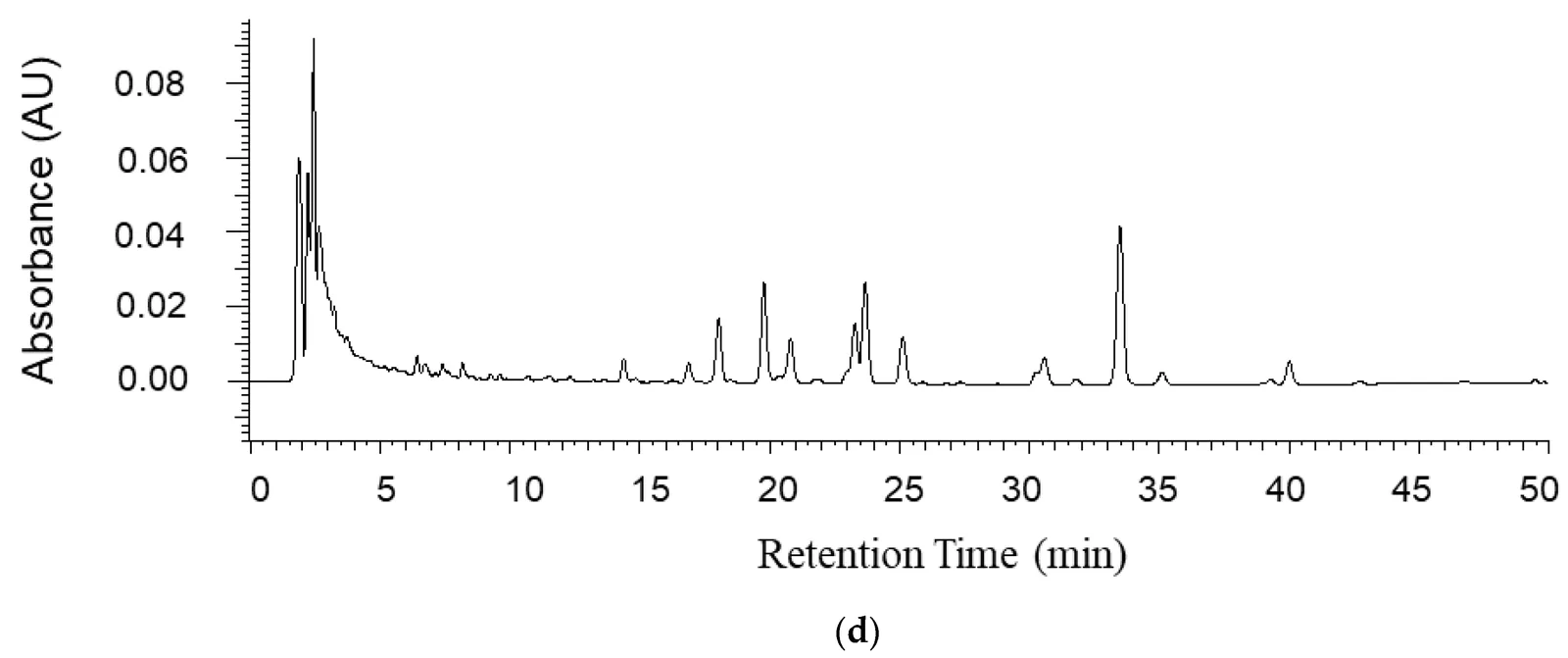
High−performance liquid chromatography (HPLC) chromatograms of different parts of Poria Cum Pini Radix: (**a**) whole Poria Cum Pini Radix (PPR); (**b**) surface part (PPR−P); (**c**) inner part beside the surface and pine root (PPR−F); (**d**) pine root part (PPR−R). Chromatograms of PAB, DTUA, PAA, PPAC, DTRA, and DEA of four parts. The UV detector was set to 242 nm.

**Figure 10:**
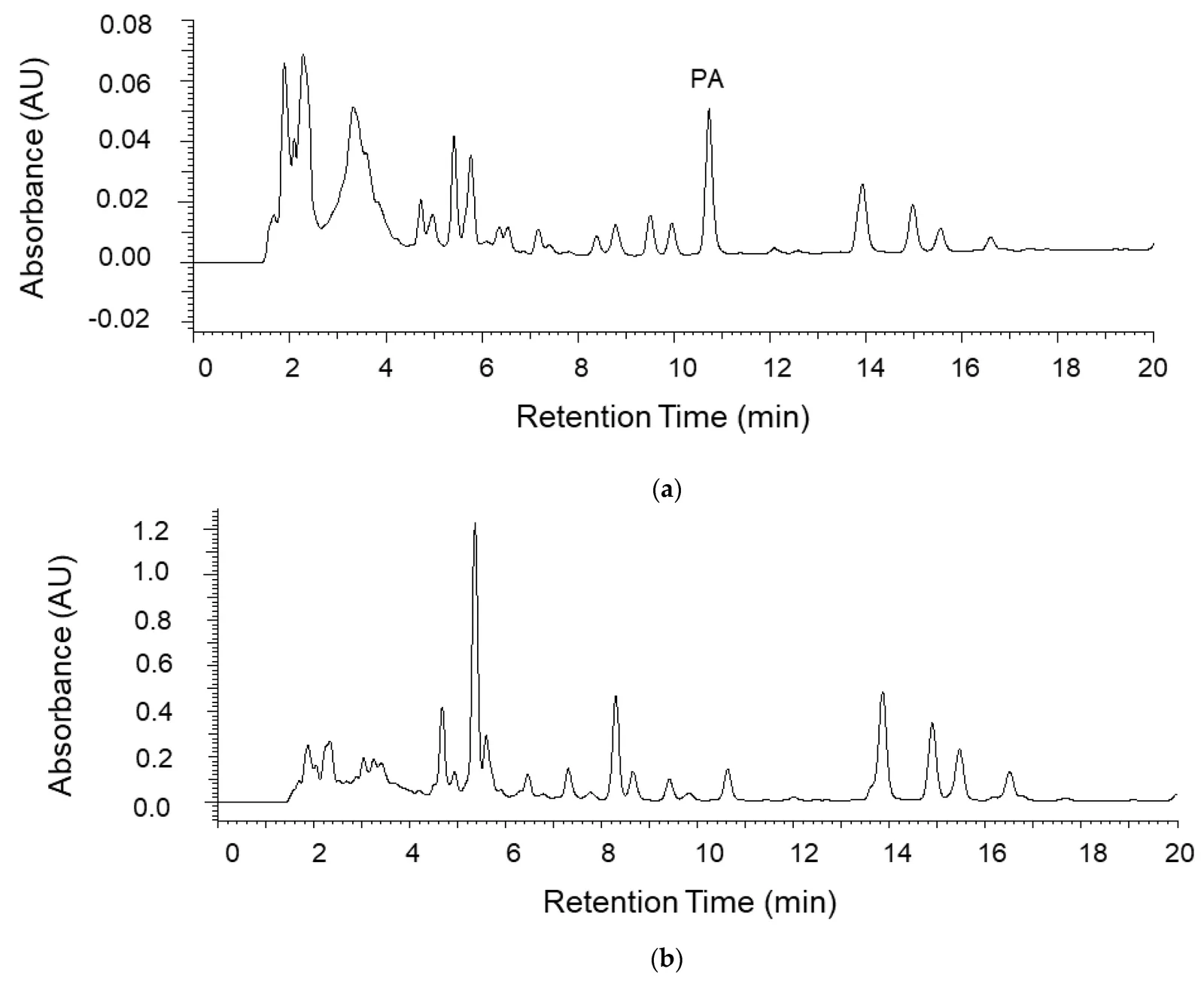

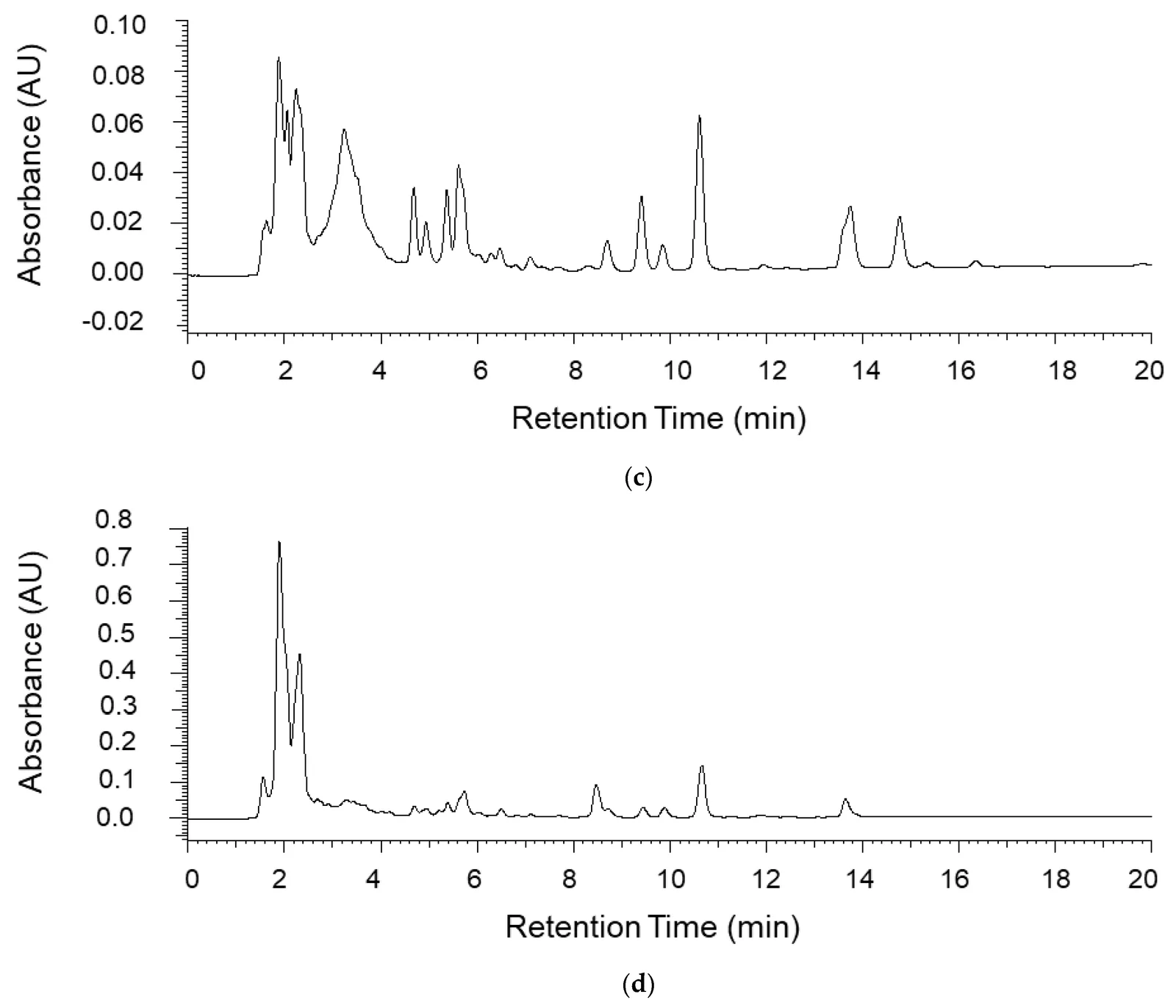
High−performance liquid chromatography (HPLC) chromatograms of different parts of Poria Cum Pini Radix: (**a**) whole Poria Cum Pini Radix (PPR); (**b**) surface part (PPR−P); (**c**) inner part beside the surface and pine root (PPR−F); (**d**) pine root part (PPR−R). Chromatograms of PA of four parts. The UV detector was set to 203 nm.

The contents of the chemical indicators were further compared among different regions of PPR. PPR was divided into three parts: PPR-P (surface), PPR-F (inner tissue between the surface and pine root), and PPR-R (pine root) (Figure 1c). Representative HPLC chromatograms of the six chemical indicators are shown in Figure 9, whereas the HPLC chromatogram obtained using the optimized method for PA determination is shown in Figure 10. The total content of the quantified triterpenoids was highest in PPR-P (Table 4). Notably, the total triterpenoid content in PPR-R was higher than that in PPR-F (Table 4). Among the quantified compounds, DTUA, PPAC, and PA reached their highest levels in PPR-R, whereas DTRA and DEA were not detected. Despite the absence of these two compounds, several characteristic triterpenoids were detected in PPR-R.

### 3.9. Network Pharmacology of the Principal Chemical Composition in WE

**Figure 11:**
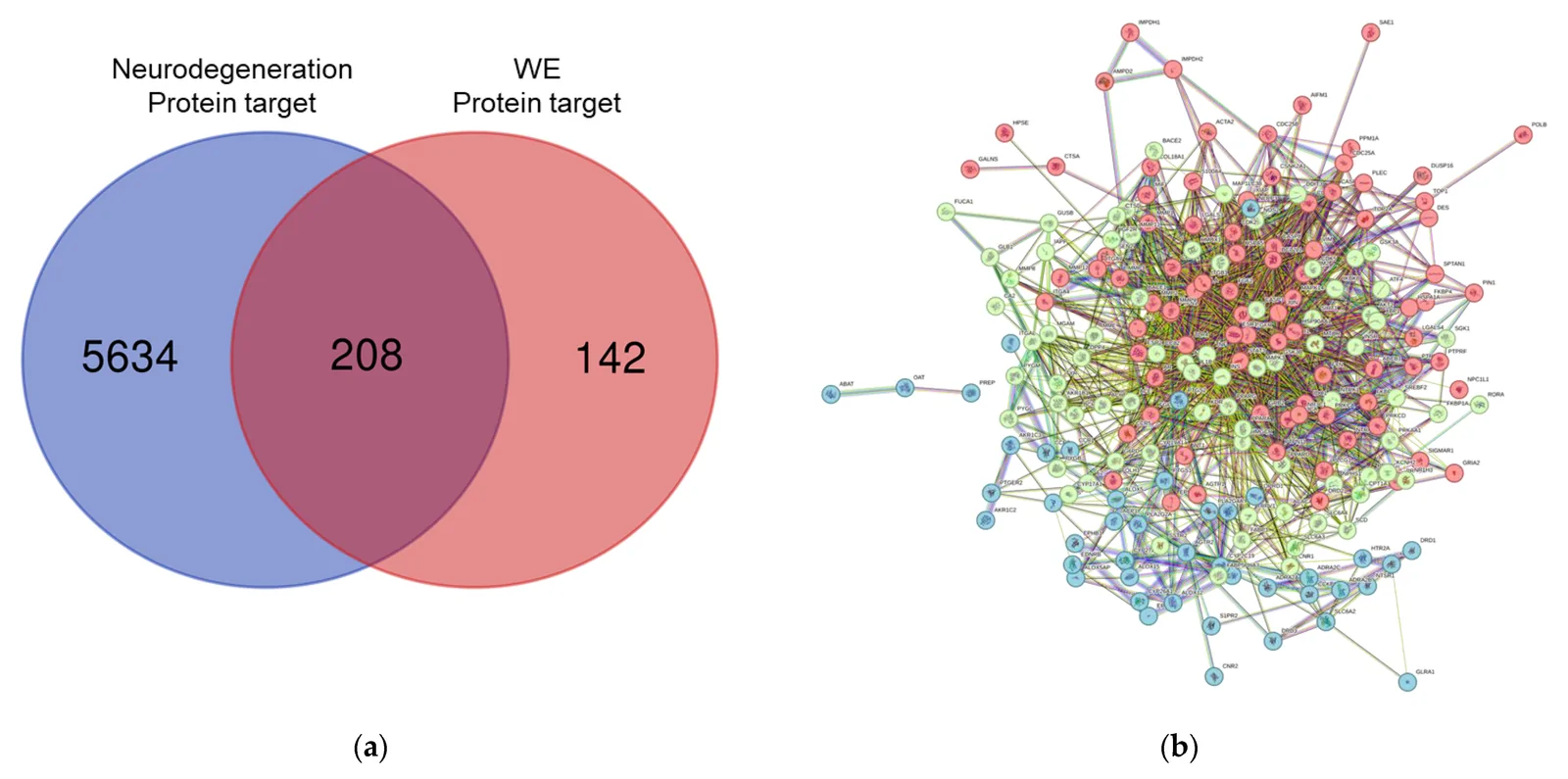

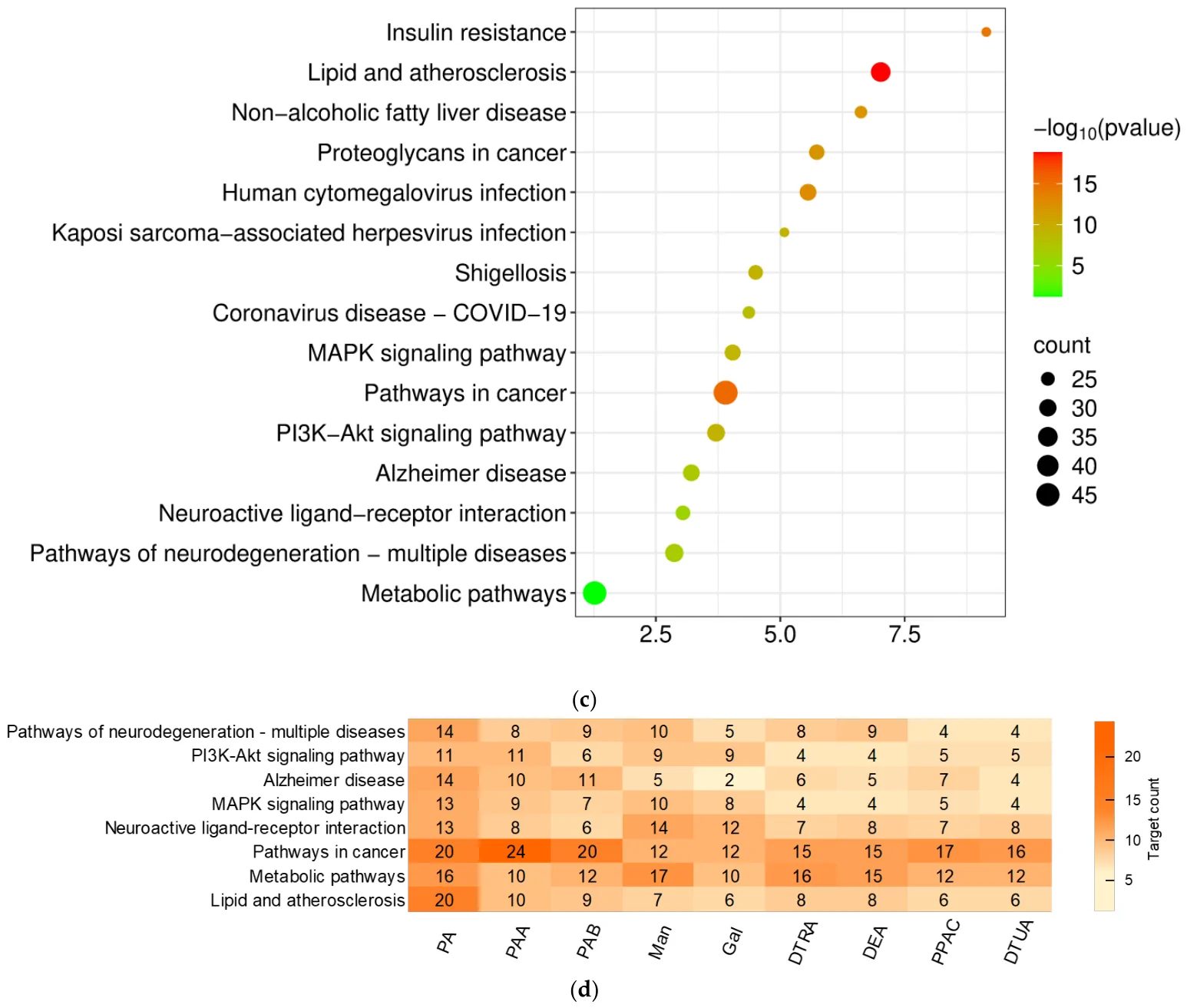
Network pharmacology from WE and neurodegeneration. (**a**) Venn diagram illustrating the overlapping protein targets of WE compounds and neurodegeneration-related pathways. (**b**) Protein–protein interaction (PPI) between 208 proteins. (**c**) The enrichment analysis revealed the top 15 KEGG pathways linked to protein targets modulated by nine compounds from WE. (**d**) A heat map visualized the relationships between nine compounds from WE and their associated target proteins involved in the top five pathway counts and neurodegeneration-related pathways. The intensity of the color gradient represents the strength of the interaction.

Nine representative compounds from WE (PAB, DTUA, PAA, PA, PPAC, DTRA, DEA, Man, and Gal) were analyzed using Swiss Target Prediction and PubChem, yielding 350 putative protein targets. In parallel, the GeneCards database retrieved 5842 genes associated with neuroprotection. The intersection of these two datasets revealed 208 common targets (Figure 11a). The PPI network constructed from these 208 targets showed extensive interactions among the predicted targets (Figure 11b). The KEGG pathway enrichment further revealed that, in addition to classical neurodegeneration-related pathways (e.g., Alzheimer’s disease, mitogen-activated protein kinase (MAPK), PI3K–Akt, and neuroactive ligand–receptor interactions), several cancer- and metabolism-related pathways were significantly enriched (Figure 11c). Compound–target–pathway network analysis further showed that PA, PAA, and PAB were connected with multiple neuro-related pathways (Figure 11d). Among the nine compounds evaluated, PA, PAA, and PAB were predicted to be potentially associated with neuro-related pathways.

## 4. Discussion

This study systematically compared the four medicinal parts of WE using chemical profiling, biological evaluation, and computational analysis. The results demonstrated distinct chemical compositions and neuroprotective activities among the medicinal parts, supporting the concept that their traditional classifications are associated with measurable chemical and biological differences. Different extraction systems were intentionally employed to serve distinct analytical purposes within an integrated quality evaluation strategy for WE. Aqueous extracts were prepared to reflect the traditional decoction form commonly used in clinical practice and therefore provide biologically relevant information for functional evaluation. In contrast, methanol extracts were used for comprehensive chemical profiling and quantification of triterpenoids, as these hydrophobic constituents are poorly soluble in water but are important chemical markers for quality assessment and chemotaxonomic differentiation. By integrating triterpenoid profiling, monosaccharide analysis, network pharmacology, and statistical modeling, this study further proposes a practical quality evaluation framework for differentiating the four medicinal parts.

Although all four medicinal parts of WE have been traditionally used clinically, their comparative neuroprotective activities have rarely been investigated. In the present study, the aqueous extracts exhibited distinct protective effects across three complementary in vitro models representing oxidative stress, neuroinflammation, and demyelination-related injury. Among the four medicinal parts, WP consistently showed superior protection against H_2_O_2_-induced neuronal injury and LPC-induced oligodendrocyte damage, whereas the remaining medicinal parts displayed comparatively weaker or model-dependent activities. These findings suggest that the traditional classification of WE medicinal parts is associated not only with morphological differences but also with distinct biological functions. Because only aqueous extracts were evaluated in this study, the observed differences are likely attributable to variations in water-soluble constituents, particularly polysaccharides and other hydrophilic metabolites, rather than triterpenoids, which were not detected in the extracts.

To explore the chemical basis underlying these biological differences, the monosaccharide composition of the aqueous extracts was compared among the four medicinal parts. Although Glc was the predominant monosaccharide in all samples, WP contained relatively higher levels of Man and Gal than the other medicinal parts, consistent with previous reports describing higher concentrations of these monosaccharides in fungal mycelia than in mature sclerotia [5,14,55]. Because the D-mannose/L-galactose pathway is the primary route of ascorbate production in plants [56], which has been associated with antioxidant and neuroprotective activities, the enrichment of these monosaccharides may partially explain the superior neuroprotective effects observed for WP. These differences may reflect variations in polysaccharide composition among the medicinal parts. However, because no structural characterization, such as NMR spectroscopy or molecular weight distribution analysis, was performed, it remains unclear whether the detected monosaccharides originated from specific polysaccharide backbones (e.g., β-pachyman) or represented degradation-derived components. However, the relatively strong protective effect of PC in the LPC-induced demyelination model could not be explained by monosaccharide composition alone, suggesting that additional bioactive constituents contribute to the distinct biological activities of the four medicinal parts. Therefore, further characterization of triterpenoid composition was undertaken to identify complementary chemical markers associated with medicinal-part differentiation.

**Figure 12:**
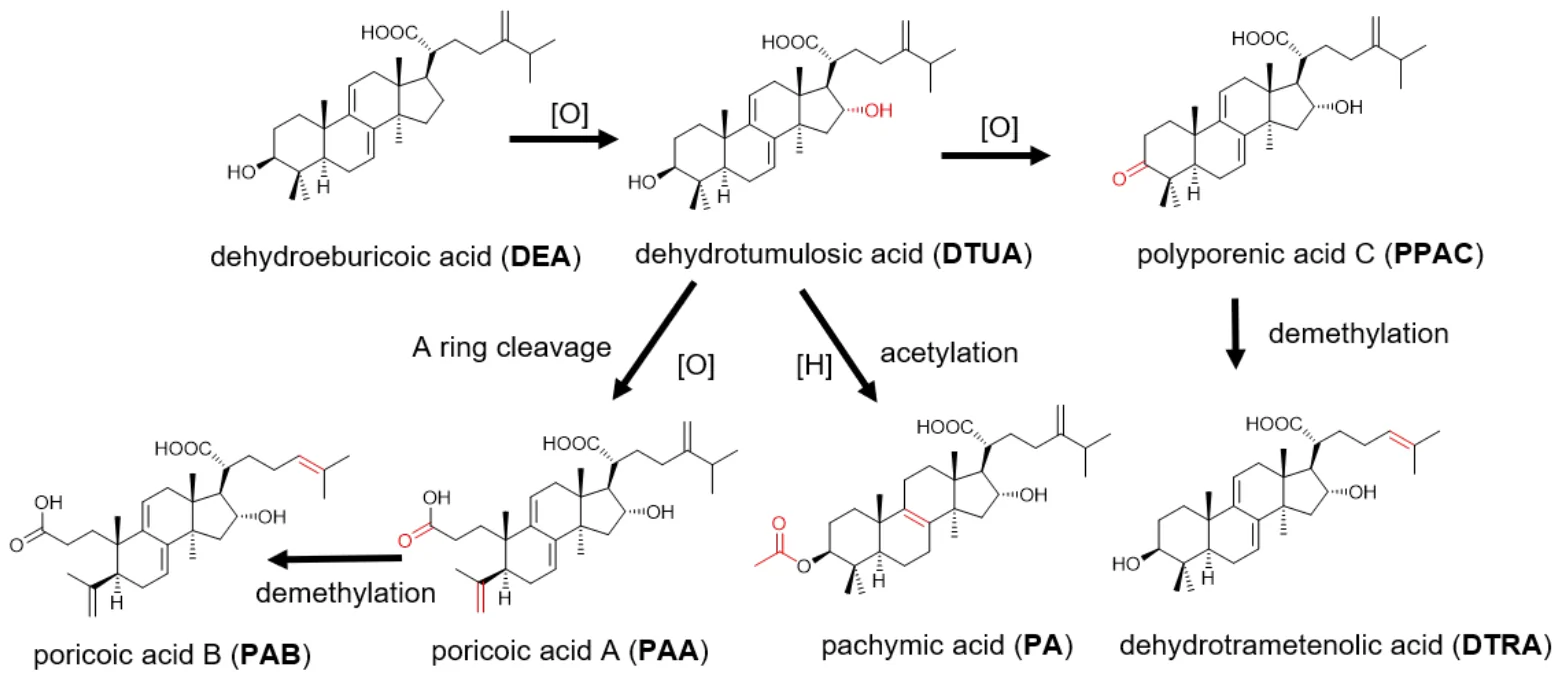
Biosynthesis hypothesis of WE triterpenoids.

Because monosaccharide composition alone could not fully account for the observed biological differences, representative triterpenoids were further investigated. The seven selected triterpenoids exhibited distinct distribution patterns among the four medicinal parts, indicating tissue-specific metabolic characteristics. In particular, WP and RP were enriched in upstream triterpenoid intermediates, whereas PC contained higher levels of downstream metabolites, including PAA, DTRA, and PAB. These findings are consistent with previous reports showing that lanostane-type triterpenoids accumulate preferentially in the outer layers of natural sclerotia, whereas earlier biosynthetic intermediates are relatively enriched in the inner tissues [22,57,58]. The present study further demonstrates that these spatial metabolic differences can be translated into practical chemical markers for distinguishing the four medicinal parts. These distribution patterns are consistent with the proposed biosynthetic pathway in which DEA serves as a precursor that undergoes sequential hydroxylation, acetylation, demethylation, and A-ring cleavage to generate structurally diverse downstream products (Figure 12). Such tissue-dependent metabolic conversion provides a biochemical explanation for the distinct chemical characteristics of the four medicinal parts and supports the use of triterpenoid profiles as reliable indicators for authentication and quality evaluation.

Based on the distinct triterpenoid distribution patterns observed among the four medicinal parts, the suitability of currently used pharmacopeial quality markers was further evaluated. According to the THP4th, PPAC is designated as the reference compound for PC, whereas PA is used as the quality marker for WP, RP, and PPR. However, the present quantitative results indicate that these markers do not adequately reflect the chemical characteristics of each medicinal part. Specifically, PAA was the most abundant triterpenoid in PC, DTUA was predominant in WP, and PA remained the major constituent in RP and PPR. These findings suggest that the current single-marker system may not fully capture the chemical diversity across different medicinal parts of WE. Analysis of commercially available samples revealed the presence of two forms of both RP and PPR. Samples containing the outer cutis consistently exhibited higher contents of the analyzed chemical constituents than those without the cutis. Moreover, color difference analysis showed that PPR samples without the cutis had higher L* values, reflecting their lighter appearance. Therefore, the use of part-specific chemical indicators, including PAA, DTUA, and PA, may provide a more accurate and representative approach for quality evaluation and authentication of WE.

To further support the biological relevance of the identified chemical markers, a network pharmacology approach was employed to explore the potential neuroprotective mechanisms of representative triterpenoids. The results indicated that nine compounds were associated with 208 neuroprotection-related targets, suggesting broad pharmacological interactions. Among these, PA, PAA, and PAB were most frequently implicated in key neurodegenerative disease-related pathways, including Alzheimer’s disease, PI3K–Akt signaling, and MAPK signaling pathways. Although these interactions were derived from computational predictions rather than experimental validation, they provide a systems-level rationale supporting the biological relevance of the proposed chemical markers. Consistent with this interpretation, previous studies have demonstrated the neuroprotective activity of PA [59,60]. In contrast, while direct evidence for the neuroprotective effects of PAA and PAB is still limited, these compounds have been reported to participate in signaling pathways associated with neurological function, supporting their potential involvement in the neuroprotective properties of WE [33,61,62,63]. In addition, the PA/PAB ratio exhibited clear variation among the four medicinal parts, suggesting its potential utility as an additional chemical index for sample discrimination. Collectively, these findings bridge chemical profiling with predicted biological functions, thereby strengthening the rationale for selecting PA, PAA, and PAB as candidate bioactivity-associated quality markers (Q-markers).

**Figure 13:**
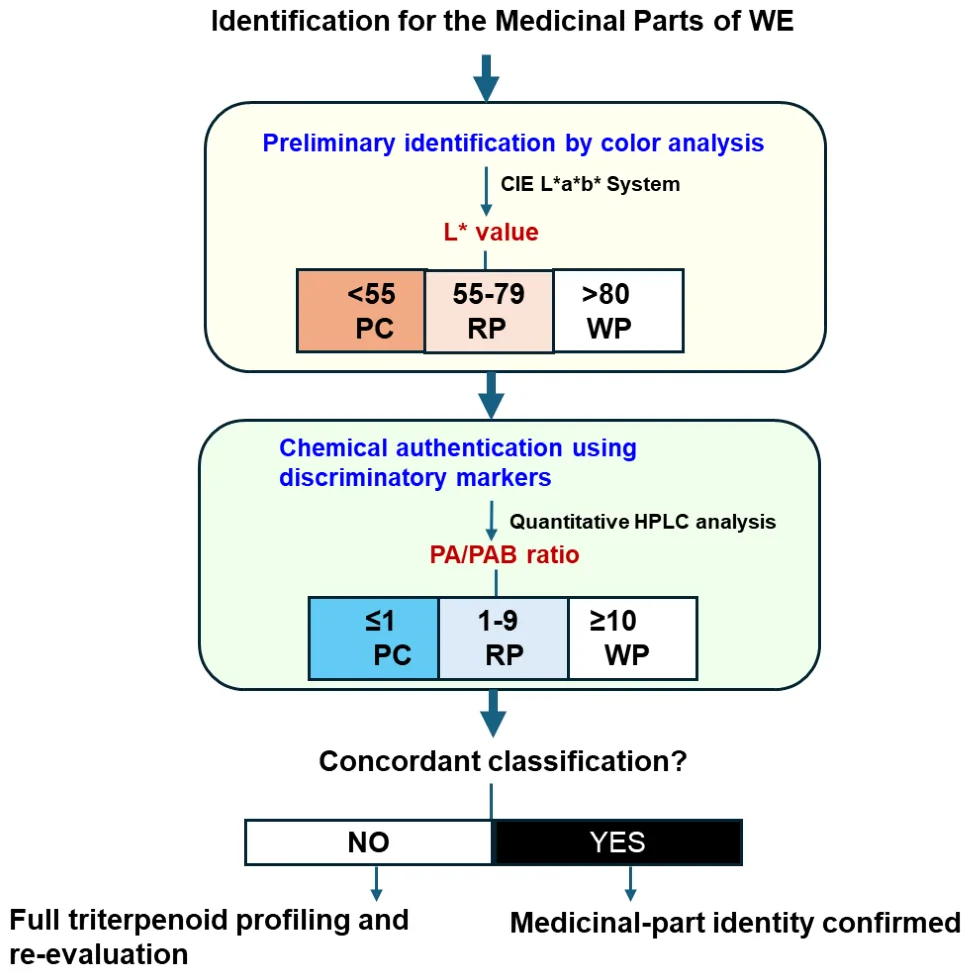
Proposed decision framework for the identification and chemical authentication of the medicinal parts of WE.

Based on the integration of comprehensive chemical profiling, multivariate statistical analysis, and biologically informed interpretation, a stepwise framework for the identification and quality evaluation of the medicinal parts of WE is proposed (Figure 13). The framework begins with preliminary identification based on the CIE L*a*b* color system. The L* value provides an objective color parameter for distinguishing PC (L* < 55), RP (55 ≤ L* < 80), and WP (L* ≥ 80). Chemical authentication is subsequently performed by quantitative HPLC analysis using the PA/PAB ratio as a discriminatory marker. PA/PAB ratios of <1, 1 to <10, and ≥10 are proposed for classifying PC, RP, and WP, respectively. Concordant classification based on the L* value and PA/PAB ratio supports confirmation of medicinal-part identity, whereas discordant results require further evaluation using the complete seven-triterpenoid profile. Thus, the proposed decision framework provides explicit analytical inputs, classification thresholds, and decision outputs for the objective and reproducible identification of PC, RP, and WP.

PPR could not be reliably distinguished using this decision system because its chemical characteristics substantially overlapped with those of RP (Figure 7) and because commercial PPR materials may be prepared either with or without the cutis. Therefore, the identification of PPR should also consider its defining macroscopic characteristic: the presence of an embedded pine root. This limitation also indicates that chemical classification alone may not be sufficient for all traditional medicinal parts of WE and highlights the importance of integrating macroscopic and chemical identification criteria.

Following medicinal-part identification, part-specific high-content triterpenoids may provide targeted chemical indicators for quality assessment. DTUA, PA, and PAA were identified as representative high-content compounds in WP, RP, and PC, respectively, and are therefore proposed as candidate assay markers for part-specific quality evaluation. In parallel, network pharmacology provided complementary evidence for the potential neuroprotection-related biological relevance of selected triterpenoids. The biological evaluation of aqueous extracts further demonstrated differences in neuroprotective activity among the medicinal parts. Higher levels of PA, PAA, PAB mannose, and galactose were associated with extracts exhibiting stronger neuroprotective effects. These findings provide a function-oriented context for interpreting chemical differences among WE medicinal parts, rather than serving as direct, routine classification criteria.

Compared with conventional single-marker quality control strategies, the proposed framework adopts a hierarchical approach that integrates preliminary color-based identification, quantitative chemical authentication, part-specific chemical indicators, and biologically informed interpretation. This strategy provides a more systematic and reproducible basis for the authentication and quality assessment of WE medicinal materials, while distinguishing practical routine identification criteria from supporting evidence for the biological relevance of candidate quality markers.

## 5. Conclusions

We established a quality evaluation framework for WE by integrating chemical profiling, bioactivity assays, and network pharmacology. Triterpenoids DTUA, PA, and PAA can be used as chemical indicators in WP, RP, and PC. Among the monosaccharides, Man and Gal demonstrated significant differences between the four medicinal components. Correspondingly, the WP aqueous extract protected SH-SY5Y cells against oxidative stress, and the PC aqueous extract reduced LPC-induced damage in HOG cells. PA, PAA, and PAB were identified as candidate bioactivity-associated Q-markers associated with neuroprotective pathways, whereas PA/PAB and L* values served as reliable identification markers to discriminate between WP, RP, and PC. These findings provide scientific evidence for the standardization, authentication, and rational use of WE in traditional medicine as well as for the development of functional foods.

## Figures and Tables

**Table 1:** Validation data for the quantitative analysis of seven analytes.

Analyte	Calibration Curve	*r* ^2 a^	Linear Range (μg/mL)	LOD ^b^ (μg/mL)	LOQ ^c^ (μg/mL)
PAB	y = 7357.3x + 1323	0.9999	1.8–250	2.03	6.77
DTUA	y = 4053.8x − 2739.5	0.9998	15.6–250	0.54	1.80
PAA	y = 7719.8x − 24049	0.9993	7.8–125	4.23	14.09
PPAC	y = 10702x − 82916	0.9994	7.8–500	11.59	38.63
PA	y = 4216.5x + 9125.9	0.9999	7.8–250	1.19	3.98
DTRA	y = 10695x + 21487	0.9999	15.6–500	1.93	6.42
DEA	y = 8583x − 1085.6	0.9990	15.6–500	2.45	8.15

^a^ *r*^2^, coefficient of determination, ^b^ LOD, limit of detection; ^c^ LOQ, limit of quantification.

**Table 2:** Precision and recovery data for the quantitative determination of seven analytes.

Analyte	Precision ^a^		Accuracy ^b^		
	Intra-Day (RSD, %)	Inter-Day (RSD, %)	Spiked Amount (μg/mL)	Recovery ^b^ (%, Mean ± SD ^c^)	RSD (%) ^d^
PAB	2.88	1.59	7.8	101.42 ± 5.01	4.94
			15.6	99.36 ± 5.28	5.32
DTUA	2.57	2.32	15.6	109.98 ± 2.15	1.95
			62.5	107.83 ± 3.18	2.95
PAA	2.36	1.30	15.6	102.36 ± 3.75	3.67
			31.25	100.78 ± 3.97	3.94
PPAC	0.58	2.53	15.6	99.88 ± 5.88	5.89
			31.25	100.48 ± 0.84	0.84
PA	3.61	3.79	15.6	93.86 ± 1.09	2.02
			31.25	96.83 ± 1.61	1.67
DTRA	2.81	1.26	31.25	102.39 ± 0.86	0.84
			62.5	101.36 ± 5.98	5.90
DEA	2.46	1.43	31.25	98.43 ± 7.04	7.52
			62.5	101.36 ± 5.98	5.90

^a^ Intra-day (*n* = 6); inter-day (*n* = 3); ^b^ Recovery (%) = [(original amount − detected amount)/spiked amount] × 100; ^c^ SD, standard deviation; ^d^ RSD, relative standard deviation.

**Table 3:** Contents of seven analytes in different WE samples.

Analyte	WP	RP	PC
PAB	0.05 ± 0.00 ^b^	0.12 ± 0.00 ^b^	7.21 ± 0.65 ^a^
DTUA	0.53 ± 0.03 ^b^	0.47 ± 0.02 ^b^	3.19 ± 0.36 ^a^
PAA	0.05 ± 0.00 ^b^	0.08 ± 0.00 ^b^	11.55 ± 1.25 ^a^
PPAC	0.07 ± 0.00 ^b^	0.09 ± 0.00 ^b^	1.05 ± 0.12 ^a^
PA	0.48 ± 0.04 ^b^	0.87 ± 0.07 ^b^	2.24 ± 0.27 ^a^
DTRA	ND	0.10 ± 0.01 ^b^	7.52 ± 0.96 ^a^
DEA	ND	0.03 ± 0.01 ^b^	5.99 ± 0.73 ^a^
Total *	1.13 ± 0.02 ^b^	1.76 ± 0.05 ^b^	34.42 ± 3.23 ^a^

Units: mg/g. WP, white part of one sclerotium; RP, pink part of one sclerotium; PC, surface part of one sclerotium. * Total content of the seven analytes. Data are presented as mean ± SD (*n* = 3). Different superscript letters within the same row indicate significant differences among the medicinal parts (one-way ANOVA followed by Tukey’s multiple comparison test, *p* < 0.05).

**Table 4:** Contents of seven analytes in different parts of PPR.

Analyte	PPR-P	PPR-F	PPR-R
PAB	2.25 ± 0.03 ^a^	0.16 ± 0.00 ^b^	0.15 ± 0.01 ^b^
DTUA	1.40 ± 0.00 ^a^	0.18 ± 0.01 ^b^	0.44 ± 0.05^c^
PAA	9.59 ± 0.14 ^a^	0.20 ± 0.02 ^b^	0.15 ± 0.04 ^b^
PPAC	0.74 ± 0.04 ^a^	0.14 ± 0.00 ^b^	0.20 ± 0.01 ^b^
PA	1.76 ± 0.13 ^a^	0.77 ± 0.06 ^b^	1.71 ± 0.03 ^a^
DTRA	4.74 ± 0.01 ^a^	0.19 ± 0.00 ^b^	ND
DEA	4.15 ± 0.01 ^a^	0.04 ± 0.00 ^b^	ND
Total *	24.65 ± 0.00 ^a^	1.68 ± 0.03 ^b^	2.65 ± 0.08 ^c^

Units: mg/g. PPR−P, surface part of Poria Cum Pini Radix; PPR−F, inner part beside the surface and pine root of Poria Cum Pini Radix; PPR−R, pine root of Poria Cum Pini Radix. * Total content of the seven analytes. Data are presented as mean ± SD (*n* = 3). Different superscript letters within the same row indicate significant differences among the medicinal parts (one-way ANOVA followed by Tukey’s multiple comparison test, *p* < 0.05).

## Data Availability

The original contributions of this study are included in this article. Further inquiries can be directed to the corresponding author.

## Supplementary Materials

The following supporting information can be downloaded at: https://www.mdpi.com/article/10.3390/life16081241/s1, Figure S1: High-performance liquid chromatograph (HPLC) chromatograms of monosaccharide. Man, mannose; Rib, ribose; Rha, rhamnose; GlcNAc, N-acetylglucosamine; GlcA, glucuronic acid; GalA, galacturonic acid; Glc, glucose; GalNAc, N-acetylgalactosamine; Gal, galatose; Xyl, xylose; Ara, arabinose; Fuc, fucose, Figure S2: High-performance liquid chromatograph (HPLC) chromatograms of seven chemical indicators. PAB, poricoic acid B; DTUA, dehydrotumulosic acid; PAA, poricoic acid A; PPAC, polyporenic acid C; PA, pachymic acid; DTRA, dehydrotrametenolic acid; DEA, dehydroeburicoic acid. Separation used a LiChrospher100 RP-18 endcapped column (4 mm i.d. × 250 mm, 5 μm; Merck) at a column oven temperature of 40 °C and a flow rate of 1.0 mL/min. (a) The mobile phase consisted of A (0.05% trifluoroacetic acid) and B (acetonitrile): 0~5 min, 40%~50% B; 5~30 min, 50%~70% B; 30~50 min, 70%~100% B; 51 min, 40% B; 51~60 min, 40% B. The UV detector was set to 242 nm. (b) The mobile phase consisted of A (0.1 % phosphoric acid) and B (acetonitrile): 0~20 min, 70%~100% B. The UV detector was set to 203 nm, Table S1: Specimens of different parts of *Wolfiporia extensa*, Table S2: Contents of seven analytes in *Wolfiporia extensa* samples obtained from the markets, Table S3: Color parameters in the CIE L*a*b* system for *Wolfiporia extensa* samples obtained from the markets, Table S4: Extraction Yield (%) of 22 *Wolfiporia extensa* samples (water extract), Table S5. Monosaccharide composition and molar proportion (%) of 22 *Wolfiporia extensa* samples.

## Abbreviations

The following abbreviations are used in this manuscript:

Ara	Arabinose
ChP^2025^	Chinese Pharmacopeia 2025 edition
CIE	International Commission on Illumination
DAD	Diode array detection
DEA	Dehydroeburicoic acid
DTRA	Dehydrotrametenolic acid
DTUA	Dehydrotumulosic acid
Fuc	Fucose
Gal	Galactose
GalA	Galacturonic acid
GalNAc	N-Acetylgalactosamine
Glc	Glucose
GlcA	Glucuronic acid
GlcNAc	N-Acetylglucosamine
HOG cells	Human oligodendroglioma cells
HPLC	High-performance liquid chromatography
KEGG	Kyoto encyclopedia of genes and genomes
LDA	Linear discriminant analysis
LOD	Limits of detection
LOQ	Limits of quantification
LPC	L-Alpha lysophosphatidylcholine
LPS	Lipopolysaccharide
Man	Mannose
NO	Nitric oxide
PA	Pachymic acid
PAA	Poricoic acid A
PAB	Poricoic acid B
PC	Poriae Cutis
PMP	1-Phenyl-3-methyl-5-pyrazolone
PPAC	Polyporenic acid C
PPI	Protein–protein interactions
PPR	Poria cum Pini Radix
PS	Streptomycin and penicillin
Q-marker	Quality markers
Rha	Rhamnose
Rib	Ribose
RP	Rubra Poria
RSDs	Relative standard deviations
TCM	Traditional Chinese Medicine
TFA	Trifluoroacetic acid
THP4th	Fourth edition of the Taiwan Herbal Pharmacopeia
WE	*Wolfiporia extensa* (Peck) Ginns
WP	Poria
Xyl	Xylose
