# Supplementary material for: Integrated Chemical Profiling and Network Pharmacology Establish a Quality Evaluation Framework for the Four Medicinal Parts of Wolfiporia extensa (Peck) Ginns
Source: Life (Basel). 2026 Jul 27;16(8):1241. doi: 10.3390/life16081241 (PMC13514367; doi:10.3390/life16081241)
Supplement: Supplementary file 1 [file life-16-01241-s001.zip › life-4370499-supplementary.pdf]

Supplementary Materials

Integrated Chemical Profiling and Network Pharmacology Establish a Quality Evaluation Framework for the Four Medicinal Parts of *Wolfiporia extensa* (Peck) Ginns

Shun-Xin Deng, Lih-Geeng Chen, Shih-Yi Hsiung, Vinh Tuyen T. Le, Chia-Jung Lee, Yves S. Y. Hsieh, and Ching-Chiung Wang

Supplementary Materials

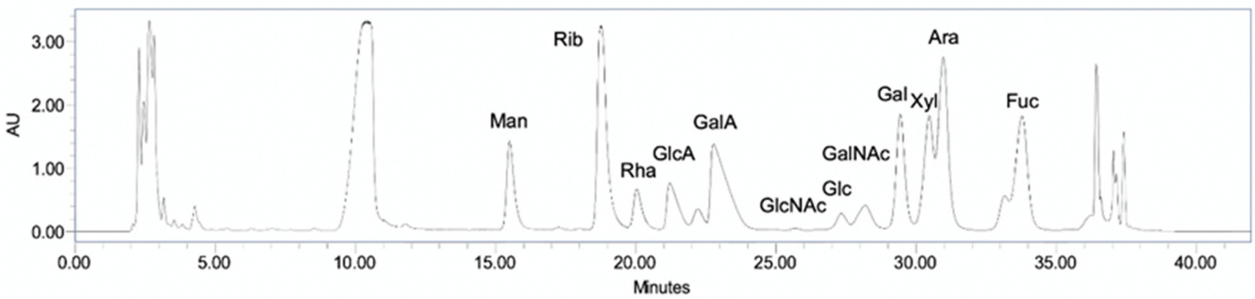

Figure S1. High-performance liquid chromatograph (HPLC) chromatograms of monosaccharide. Man, mannose; Rib, ribose; Rha, rhamnose; GlcNAc, N-acetylglucosamine; GlcA, glucuronic acid; GalA, galacturonic acid; Glc, glucose; GalNAc, N-acetylgalactosamine; Gal, galatose; Xyl, xylose; Ara, arabinose; Fuc, fucose.

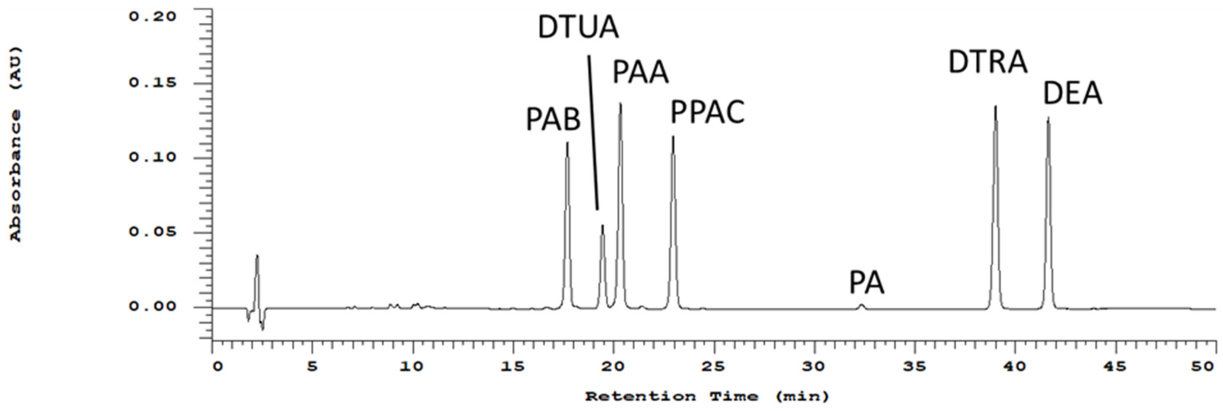

(**a**)

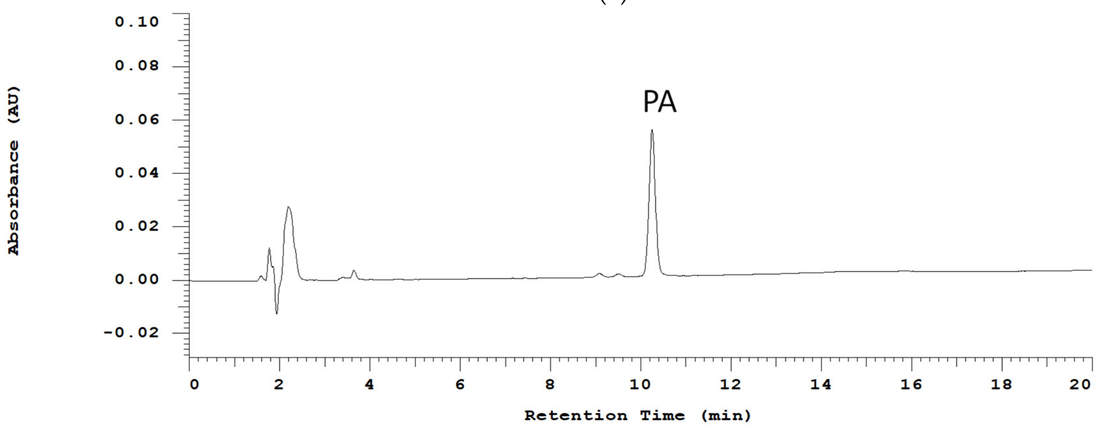

(**b**)

Figure S2. High-performance liquid chromatograph (HPLC) chromatograms of seven chemical indicators. PAB, poricoic acid B; DTUA, dehydrotumulosic acid; PAA, poricoic acid A; PPAC, polyporenic acid C; PA, pachymic acid; DTRA, dehydro-trametenolic acid; DEA, dehydroeburicoic acid. Separation used a LiChrospher100 RP-18 endcapped column (4 mm i.d. × 250 mm, 5 μm; Merck) at a column oven temperature of 40 °C and a flow rate of 1.0 mL/min. (a) The mobile phase consisted of A (0.05 % trifluoro-acetic acid) and B (acetonitrile): 0~5 min, 40%~50% B; 5~30 min, 50%~70% B; 30~50 min, 70%~100% B; 51 min, 40% B; 51~60 min, 40% B. The UV detector was set to 242 nm. (b) The mobile phase consisted of A (0.1 % phosphoric acid) and B (acetonitrile): 0~20 min, 70%~100% B. The UV detector was set to 203 nm.

Table S1. Specimens of different parts of *Wolfiporia extensa.*

Sample code	Used part	Collection time	Locality
WP-1	Poria	July 2019	Taiwan
WP-2	Poria	July 2019	Taiwan
WP-3	Poria	July 2019	Taiwan
WP-4	Poria	July 2019	Taiwan
WP-5	Poria	July 2019	Taiwan
WP-6	Poria	July 2019	Taiwan
WP-7	Poria	July 2019	Taiwan
WP-8	Poria	July 2019	Taiwan
WP-9	Poria	July 2019	Taiwan
WP-10	Poria	July 2019	Taiwan
WP-11	Poria	July 2019	Taiwan
WP-12	Poria	July 2019	Taiwan
WP-13	Poria	July 2019	Taiwan
WP-14	Poria	July 2019	Taiwan
WP-15	Poria	July 2019	Taiwan
WP-16	Poria	July 2019	Taiwan
WP-17	Poria	July 2019	Taiwan
WP-18	Poria	July 2019	Taiwan
WP-19	Poria	July 2019	Taiwan
WP-20	Poria	July 2019	Taiwan
WP-21	Poria	Aug. 2021	Taiwan
WP-22	Poria	Aug. 2021	Taiwan
WP-23	Poria	Aug. 2021	Taiwan
WP-24	Poria	Aug. 2021	Taiwan
WP-25	Poria	Aug. 2021	Taiwan
WP-26	Poria	May 2019	Hunan, China
WP-27	Poria	May 2019	Hunan, China
WP-28	Poria	May 2019	Hunan, China
WP-29	Poria	Oct. 2019	Anhui, China
WP-30	Poria	Oct. 2019	Anhui, China
RP-1	Rubra Poria	Aug. 2021	Taiwan
RP-2	Rubra Poria	Aug. 2021	Taiwan
RP-3	Rubra Poria	Aug. 2021	Taiwan
RP-4	Rubra Poria	Aug. 2021	Taiwan
RP-5	Rubra Poria	Aug. 2021	Taiwan
RP-6	Rubra Poria	Aug. 2021	Taiwan
RP-7	Rubra Poria	May 2019	Hunan, China
RP-8	Rubra Poria	May 2019	Hunan, China
RP-9	Rubra Poria	Oct. 2019	Anhui, China
RP-10	Rubra Poria	Aug. 2021	Anhui, China

RP-11	Rubra Poria	Aug. 2021	Yunnan, China
PC-1	Poriae Cutis	Mar. 2019	Taiwan
PC-2	Poriae Cutis	Aug. 2021	Taiwan
PC-3	Poriae Cutis	Aug. 2021	Taiwan
PC-4	Poriae Cutis	Aug. 2021	Taiwan
PC-5	Poriae Cutis	Aug. 2021	Taiwan
PC-6	Poriae Cutis	Aug. 2021	Taiwan
PC-7	Poriae Cutis	Aug. 2021	Anhui, China
PC-8	Poriae Cutis	Aug. 2021	Yunnan, China
PPR-1	Poria Cum Pini Radix	Aug. 2021	Taiwan
PPR-2	Poria Cum Pini Radix	Aug. 2021	Taiwan
PPR-3	Poria Cum Pini Radix	Aug. 2021	Taiwan
PPR-4	Poria Cum Pini Radix	Aug. 2021	Taiwan
PPR-5	Poria Cum Pini Radix	Aug. 2021	Taiwan
PPR-6	Poria Cum Pini Radix	Aug. 2021	Taiwan
PPR-7	Poria Cum Pini Radix	Aug. 2021	Taiwan
PPR-8	Poria Cum Pini Radix	Jan. 2019	Hunan, China
PPR-9	Poria Cum Pini Radix	May 2019	Hunan, China

Table S2. Contents of seven analytes in *Wolfiporia extensa* samples obtained from the markets.

Sample code	PAB	DTUA	PAA	PPAC	PA	DTRA	DEA	Sum
WP-1	0.10	0.22	0.08	0.18	0.91	0.02	-	1.51
WP-2	0.02	0.86	0.06	0.15	0.44	-	-	1.53
WP-3	0.04	0.71	0.07	0.17	0.58	-	-	1.57
WP-4	0.02	0.78	0.03	0.14	0.35	-	-	1.32
WP-5	0.08	0.65	0.09	0.18	0.87	0.01	-	1.88
WP-6	0.03	1.14	0.07	0.18	0.58	-	-	2.00
WP-7	0.10	0.46	0.07	0.19	0.90	0.01	-	1.73
WP-8	0.12	0.53	0.10	0.19	0.81	0.01	-	1.76
WP-9	0.08	0.51	0.07	0.18	0.86	-	-	1.70
WP-10	0.01	1.29	0.03	0.17	0.31	-	-	1.81
WP-11	0.01	0.66	0.03	0.13	0.34	-	-	1.17
WP-12	0.09	0.27	0.08	0.11	0.65	-	-	1.20
WP-13	0.03	1.05	0.05	0.20	0.65	-	-	1.98
WP-14	0.01	0.30	0.04	0.10	0.36	-	-	0.81
WP-15	0.04	1.44	0.04	0.26	0.74	-	-	2.52
WP-16	0.04	0.52	0.04	0.22	0.55	-	-	1.37
WP-17	0.09	0.27	0.08	0.18	0.87	-	-	1.49
WP-18	0.01	0.92	0.03	0.19	0.36	-	-	1.51
WP-19	0.04	0.46	0.06	0.17	0.65	-	-	1.38
WP-20	0.02	0.77	0.03	0.14	0.28	-	-	1.24
WP-21	0.01	0.55	0.03	0.15	0.46	-	-	1.20
WP-22	0.06	0.80	0.03	0.21	1.16	-	-	2.26
WP-23	0.08	0.31	0.09	0.18	0.64	-	-	1.30
WP-24	0.05	0.56	0.03	0.18	0.64	-	-	1.46
WP-25	0.03	1.11	0.03	0.20	0.65	-	-	2.02
WP-26	0.01	0.83	-	0.13	0.45	-	-	1.42
WP-27	0.05	0.43	-	0.17	0.47	-	-	1.12
WP-28	0.03	0.50	0.04	0.15	0.56	-	-	1.28
WP-29	0.04	0.33	0.07	0.15	0.52	-	0.01	1.12
WP-30	0.05	0.31	0.05	0.15	0.49	0.02	0.01	1.08
RP-1	0.08	0.50	0.29	0.30	0.36	0.06	0.10	1.69
RP-2	0.26	0.24	0.57	0.32	0.74	0.23	0.15	2.51
RP-3	0.21	0.29	0.74	0.27	0.49	0.34	0.37	2.71
RP-4	0.26	0.57	0.90	0.33	0.69	0.70	0.37	3.82
RP-5	0.13	0.48	0.42	0.34	0.60	0.24	0.26	2.47
RP-6	0.13	0.71	0.42	0.26	0.76	0.16	0.13	2.57
RP-7	0.15	0.48	0.11	0.20	1.02	0.05	0.02	2.03
RP-8	0.08	0.40	0.06	0.22	0.75	0.11	-	1.62
RP-9	0.14	0.50	0.09	0.25	0.68	0.22	0.06	1.94
RP-10	0.47	0.58	0.37	0.41	1.36	0.27	0.18	3.64
RP-11	0.05	0.44	0.11	0.30	0.64	0.15	0.04	1.73
PC-1	5.36	3.20	20.96	2.60	3.12	10.28	7.89	53.41

PC-2	1.53	1.20	6.14	0.78	1.64	4.78	2.92	18.99
PC-3	4.11	3.50	15.84	2.38	1.83	4.61	5.48	37.75
PC-4	2.68	2.68	10.39	1.35	2.52	6.24	5.44	31.30
PC-5	1.62	1.45	3.51	0.97	1.56	2.35	1.50	12.96
PC-6	2.31	2.99	8.61	1.47	2.42	7.26	5.82	30.88
PC-7	5.12	4.16	9.93	1.63	1.74	2.89	3.03	28.50
PC-8	2.10	1.63	5.26	1.05	1.14	3.59	1.95	16.72
PPR-1	0.31	0.64	0.82	0.31	0.80	0.62	0.40	3.90
PPR-2	0.07	0.52	0.28	0.17	0.43	0.46	0.25	2.18
PPR-3	0.13	0.30	0.59	0.24	0.70	0.68	0.31	2.95
PPR-4	0.56	0.48	2.30	0.25	1.08	0.73	0.56	5.96
PPR-5	0.14	0.38	0.38	0.24	1.10	0.92	0.31	3.47
PPR-6	-	0.43	0.04	0.19	0.49	0.60	0.49	2.24
PPR-7	0.31	0.30	0.85	0.22	0.76	0.32	0.32	3.08
PPR-8	0.09	0.31	0.25	0.10	0.58	0.17	0.15	1.65
PPR-9	0.08	0.48	0.08	0.22	1.22	0.33	0.04	2.45

units: mg/g. -, not detectable; WP-1 to WP-30, Poria; RP-1 to RP-11, Rubra Poria; PC-1 to PC-8, Poriae Cutis; PPR-1 to PPR-9, Poria Cum Pini Radix.

Table S3. Color parameters in the CIE L*a*b* system for *Wolfiporia extensa* samples obtained from the markets

Sample code	L*	a*	b*
White paper	87.48 ± 0.11	-2.60 ± 0.00	-2.30 ± 0.07
WP-1	86.54 ± 0.92	-2.06 ± 0.23	14.12 ± 0.60
WP-2	87.34 ± 1.13	-3.20 ± 0.07	12.28 ± 0.23
WP-3	88.86 ± 0.78	-2.72 ± 0.08	12.92 ± 0.37
WP-4	86.53 ± 1.24	-2.27 ± 0.35	13.95 ± 0.83
WP-5	84.80 ± 0.62	-1.72 ± 0.26	12.94 ± 0.54
WP-6	87.91 ± 2.27	-3.09 ± 1.24	12.23 ± 1.67
WP-7	84.34 ± 0.64	-1.16 ± 0.25	14.52 ± 0.28
WP-8	83.24 ± 1.71	-1.38 ± 0.41	14.20 ± 0.60
WP-9	81.02 ± 0.54	-0.48 ± 0.16	14.66 ± 0.13
WP-10	90.66 ± 0.46	-4.22 ± 0.15	10.36 ± 0.47
WP-11	89.82 ± 0.73	-3.98 ± 0.23	11.16 ± 0.59
WP-12	79.78 ± 0.88	-0.50 ± 0.21	14.04 ± 0.29
WP-13	88.18 ± 0.98	-2.58 ± 0.11	13.22 ± 0.56
WP-14	79.52 ± 1.53	0.14 ± 0.52	17.50 ± 0.74
WP-15	87.62 ± 0.63	-2.46 ± 0.28	13.28 ± 0.54
WP-16	89.22 ± 0.75	-3.20 ± 0.12	12.04 ± 0.38
WP-17	87.26 ± 0.82	-2.32 ± 0.13	13.58 ± 0.47
WP-18	89.50 ± 0.94	-3.16 ± 0.57	12.18 ± 0.65
WP-19	87.70 ± 0.87	-2.06 ± 0.22	14.74 ± 0.61
WP-20	87.16 ± 1.16	-2.28 ± 0.23	13.78 ± 0.55
WP-21	86.18 ± 1.03	-1.86 ± 0.27	14.92 ± 0.60
WP-22	89.16 ± 0.92	-3.70 ± 0.14	11.08 ± 0.54
WP-23	85.68 ± 1.18	-1.94 ± 0.21	13.66 ± 0.56
WP-24	90.42 ± 0.38	-3.82 ± 0.11	11.24 ± 0.42
WP-25	88.48 ± 1.08	-3.08 ± 0.16	12.10 ± 0.46
WP-26	84.82 ± 0.55	-2.66 ± 0.35	14.78 ± 0.31
WP-27	86.94 ± 0.19	-3.28 ± 0.04	12.84 ± 0.09
WP-28	84.26 ± 0.71	-2.92 ± 0.08	14.10 ± 0.28
WP-29	83.30 ± 0.48	-1.14 ± 0.17	13.96 ± 0.29
WP-30	83.02 ± 0.89	-0.80 ± 0.25	14.46 ± 0.38
RP-1	75.04 ± 0.33	0.06 ± 0.19	14.36 ± 0.35
RP-2	65.68 ± 0.89	0.82 ± 0.16	14.20 ± 0.43
RP-3	67.58 ± 2.23	1.92 ± 0.28	15.68 ± 0.29
RP-4	68.98 ± 0.87	1.46 ± 0.17	15.58 ± 0.08
RP-5	67.78 ± 0.71	1.16 ± 0.26	13.78 ± 0.29
RP-6	69.62 ± 0.83	0.60 ± 0.07	12.98 ± 0.19
RP-7	70.56 ± 0.84	1.94 ± 0.23	15.44 ± 0.34
RP-8	73.20 ± 1.66	2.14 ± 0.54	17.26 ± 0.42
RP-9	51.46 ± 0.25	3.62 ± 0.15	13.06 ± 0.21
RP-10	63.58 ± 0.92	2.70 ± 0.20	14.42 ± 1.33

RP-11	50.24 ± 0.35	3.52 ± 0.11	12.66 ± 0.17
PC-1	51.53 ± 2.06	2.20 ± 0.35	13.33 ± 0.82
PC-2	51.68 ± 0.80	2.84 ± 0.05	12.40 ± 0.19
PC-3	44.22 ± 0.81	2.82 ± 0.18	10.60 ± 0.40
PC-4	50.70 ± 0.36	3.80 ± 0.10	13.13 ± 0.21
PC-5	52.48 ± 0.23	2.34 ± 0.09	12.08 ± 0.08
PC-6	55.62 ± 0.79	1.86 ± 0.15	12.72 ± 0.27
PC-7	50.60 ± 0.19	3.30 ± 0.00	12.78 ± 0.08
PC-8	49.20 ± 0.42	2.98 ± 0.08	12.04 ± 0.11
PPR-1	61.34 ± 1.46	2.44 ± 0.19	16.04 ± 0.32
PPR-2	78.48 ± 0.77	-1.42 ± 0.13	11.78 ± 0.49
PPR-3	64.74 ± 2.62	1.36 ± 0.25	14.80 ± 0.48
PPR-4	64.42 ± 3.16	1.34 ± 0.30	13.74 ± 0.44
PPR-5	64.42 ± 1.29	1.88 ± 0.56	14.48 ± 0.76
PPR-6	67.55 ± 1.04	1.35 ± 0.17	15.13 ± 0.22
PPR-7	69.74 ± 1.36	0.44 ± 0.25	13.44 ± 0.44
PPR-8	72.10 ± 0.89	0.26 ± 0.11	14.26 ± 0.18
PPR-9	81.24 ± 0.53	-2.04 ± 0.27	12.32 ± 0.51

WP-1 to WP-30, Poria; RP-1 to RP-11, Rubra Poria; PC-1 to PC-8, Poriae Cutis; PPR-1 to PPR-9, Poria Cum Pini Radix.

Table S4. Extraction Yield (%) of 22 *Wolfiporia extensa* samples (water extract).

Sample code	Extraction Yield (%)
WP-2	1.31
WP-21	1.89
WP-22	1.18
WP-25	2.94
WP-26	1.48
RP	2.80
RP-2	2.82
RP-4	12.02
RP-9	14.73
RP-10	1.90
RP-11	7.77
PC	3.28
PC-2	2.94
PC-3	4.32
PC-6	3.66
PC-7	5.77
PC-8	7.88
PPR-1	8.03
PPR-4	2.07
PPR-6	12.56
PPR-8	3.62
PPR-9	3.41

WP, Poria; RP, Rubra Poria; PC, Poriae Cutis; PPR, Poria Cum Pini Radix.

Table S5. Monosaccharide composition and mole proportion (%) of 22 *Wolfiporia extensa* samples.

Sample code	Man	GlcA	Glc	Gal	Xyl	Ara	Fuc
WP-2	4.47	0.95	72.07	7.71	0.34	4.19	10.27
WP-21	4.24	1.01	73.81	5.67	-	5.39	9.88
WP-22	9.78	2.55	61.68	12.37	0.51	1.51	11.60
WP-25	4.08	0.52	84.77	6.63	-	-	4.00
WP-26	9.34	-	71.75	11.83	0.07	-	7.01
RP	2.55	1.69	89.48	4.29	-	-	1.98
RP-2	1.07	0.97	73.49	3.97	0.83	5.11	14.56
RP-4	0.88	0.93	85.71	2.13	1.05	1.24	8.06
RP-9	0.36	0.39	95.00	1.43	0.49	0.39	1.95
RP-10	3.96	1.07	70.06	7.64	0.54	5.25	11.48
RP-11	1.93	0.42	90.95	2.99	0.42	0.41	2.88
PC	3.03	1.59	83.98	7.20	-	-	4.21
PC-2	4.64	0.52	77.40	8.15	2.22	0.47	6.60
PC-3	2.24	0.94	71.58	5.89	1.60	6.22	11.52
PC-6	7.77	0.98	59.58	11.05	2.85	3.44	14.33
PC-7	5.53	0.85	62.72	11.25	1.70	3.14	14.82
PC-8	1.82	0.47	87.65	3.70	0.96	0.96	4.44
PPR-1	-	3.68	79.67	3.38	1.72	2.34	9.21
PPR-4	-	3.81	70.18	2.53	4.42	2.84	16.22
PPR-6	3.12	1.23	67.33	9.88	0.72	6.31	11.42
PPR-8	0.53	1.72	94.81	1.97	0.08	-	0.90
PPR-9	0.50	0.82	95.54	2.17	-	-	0.97

-, not detectable; WP, Poria; RP, Rubra Poria; PC, Poriae Cutis; PPR, Poria Cum Pini Radix.
